# Small-scale field evaluation of PermaNet^®^ Dual (a long-lasting net coated with a mixture of chlorfenapyr and deltamethrin) against pyrethroid-resistant *Anopheles gambiae* mosquitoes from Tiassalé, Côte d’Ivoire

**DOI:** 10.1186/s12936-023-04455-z

**Published:** 2023-02-01

**Authors:** Julien Z. B. Zahouli, Constant A. V. Edi, Laurence A. Yao, Emmanuelle G. Lisro, Marc Adou, Inza Koné, Graham Small, Eleanore D. Sternberg, Benjamin G. Koudou

**Affiliations:** 1 Centre Suisse de Recherches Scientifiques en Côte d’Ivoire, Abidjan, Côte d’Ivoire; 2 Centre d’Entomologie Médicale et Vétérinaire, Université Alassane Ouattara, Bouaké, Côte d’Ivoire; 3 Swiss Tropical and Public Health Institute, Allschwil, Switzerland; 4 UFR Science de la Nature, Université Nagui-Abrogoua, Abidjan, Côte d’Ivoire; 5 Université Félix Houphouët-Boingy, Abidjan, Côte d’Ivoire; 6 Innovative Vector Control Consortium, Liverpool, UK; 7 Vestergaard Sàrl, Lausanne, Switzerland; 8 Department of Vector Biology, Liverpool School of Tropical Medicine, Liverpool, L3 5QA UK

**Keywords:** Malaria, *Anopheles gambiae*, Insecticide resistance, PermaNet^®^ Dual, PermaNet^®^ 3.0, PermaNet^®^ 2.0, Experimental hut, Côte d’Ivoire

## Abstract

**Background:**

Due to the rapid expansion of pyrethroid-resistance in malaria vectors in Africa, Global Plan for Insecticide Resistance Management (GPIRM) has recommended the development of long-lasting insecticidal nets (LLINs), containing insecticide mixtures of active ingredients with different modes of action to mitigate resistance and improve LLIN efficacy. This good laboratory practice (GLP) study evaluated the efficacy of the chlorfenapyr and deltamethrin-coated PermaNet^®^ Dual, in comparison with the deltamethrin and synergist piperonyl butoxide (PBO)-treated PermaNet^®^ 3.0 and the deltamethrin-coated PermaNet^®^ 2.0, against wild free-flying pyrethroid-resistant *Anopheles gambiae *sensu lato (*s.l*.), in experimental huts in Tiassalé, Côte d’Ivoire (West Africa).

**Methods:**

PermaNet^®^ Dual, PermaNet^®^ 3.0 and PermaNet^®^ 2.0, unwashed and washed (20 washes), were tested against free-flying pyrethroid-resistant *An. gambiae s.l.* in the experimental huts in Tiassalé, Côte d’Ivoire from March to August 2020. Complementary laboratory cone bioassays (daytime and 3-min exposure) and tunnel tests (nightly and 15-h exposure) were performed against pyrethroid-susceptible *An. gambiae *sensu stricto (*s.s*.) (Kisumu strain) and pyrethroid-resistant *An. gambiae s.l.* (Tiassalé strain).

**Results:**

PermaNet^®^ Dual demonstrated significantly improved efficacy, compared to PermaNet^®^ 3.0 and PermaNet^®^ 2.0, against the pyrethroid-resistant *An. gambiae s.l.* Indeed, the experimental hut trial data showed that the mortality and blood-feeding inhibition in the wild pyrethroid-resistant *An. gambiae s.l.* were overall significantly higher with PermaNet^®^ Dual compared with PermaNet^®^ 3.0 and PermaNet^®^ 2.0, for both unwashed and washed samples. The mortality with unwashed and washed samples were 93.6 ± 0.2% and 83.2 ± 0.9% for PermaNet^®^ Dual, 37.5 ± 2.9% and 14.4 ± 3.9% for PermaNet^®^ 3.0, and 7.4 ± 5.1% and 11.7 ± 3.4% for PermaNet^®^ 2.0, respectively. Moreover, unwashed and washed samples produced the respective percentage blood-feeding inhibition of 41.4 ± 6.9% and 43.7 ± 4.8% with PermaNet^®^ Dual, 51.0 ± 5.7% and 9.8 ± 3.6% with PermaNet^®^ 3.0, and 12.8 ± 4.3% and − 13.0 ± 3.6% with PermaNet^®^ 2.0. Overall, PermaNet^®^ Dual also induced higher or similar deterrence, exophily and personal protection when compared with the standard PermaNet^®^ 3.0 and PermaNet^®^ 2.0 reference nets, with both unwashed and washed net samples. In contrast to cone bioassays, tunnel tests predicted the efficacy of PermaNet^®^ Dual seen in the current experimental hut trial.

**Conclusion:**

The deltamethrin-chlorfenapyr-coated PermaNet^®^ Dual induced a high efficacy and performed better than the deltamethrin-PBO PermaNet^®^ 3.0 and the deltamethrin-only PermaNet^®^ 2.0, testing both unwashed and 20 times washed samples against the pyrethroid-susceptible and resistant strains of *An. gambiae s.l.* The inclusion of chlorfenapyr with deltamethrin in PermaNet^®^ Dual net greatly improved protection and control of pyrethroid-resistant *An. gambiae* populations. PermaNet^®^ Dual thus represents a promising tool, with a high potential to reduce malaria transmission and provide community protection in areas compromised by mosquito vector resistance to pyrethroids.

**Supplementary Information:**

The online version contains supplementary material available at 10.1186/s12936-023-04455-z.

## Background

According to the latest World Malaria Report 2022 of the World Health Organization (WHO)*,* there were 247 million malaria cases and 619,000 malaria deaths in 84 malaria endemic countries worldwide in 2021 [1, 2]. This represents about 13.4 million more cases in 2021 compared to 2019 attributable to disruptions to essential malaria services during the COVID-18 pandemic [1]. The WHO African Region, with an estimated 234 million cases in 2021, accounted for about 95% of global cases malaria [1]. The recent decline of malaria burden from 2000 to 2019 was largely due to the massive distribution and use of long-lasting insecticidal nets (LLINs) (2.5 billion LLINs were delivered from 2004 to 2021) for *Anopheles* mosquito vector control [1]. The malaria burden reduction is now slowing down and is threatened by the spread of resistance to pyrethroids (78 of 88 endemic countries have detected resistance to at least one insecticide class reported to the WHO from 2010 to 2020) [1]. The WHO Global Plan for Insecticide Resistance Management (GPIRM) has recommended the development of LLINs with insecticide mixtures of active ingredients with different modes of action to mitigate resistance [3]. The WHO Global Technical Strategy (GTS) aims for a reduction of malaria case incidence and mortality rate of at least 40% by 2020, 75% by 2025 and 90% by 2030 from the 2015 baseline [1, 4]. To meet these targets, GTS has called for the development of new tools, with combined or more effective insecticide molecules to control insecticide-resistant *Anopheles* vectors [4]. These new vector control tools must incorporate new insecticide molecules and/or insecticide mixtures containing at least two active ingredients with different modes of action for the management of malaria vector resistance to insecticides [1, 3].

Malaria is endemic throughout Côte d’Ivoire and represents the leading cause of mortality and morbidity in the country. Several studies have shown a wide spread resistance in local *Anopheles* to most of the insecticide classes currently used in malaria vector control (e.g. pyrethroids, DDT and carbamates) [5–8]. The existence of multiple mechanisms of resistance in the main vector, *Anopheles gambiae *sensu lato (*s.l*.) threatens the efficacy of vector control tools currently used in Côte d’Ivoire, including LLINs [9–11]. Meiwald et al. [11] found that pyrethroid resistance is associated with significant overexpression of *CYP6P4*, *CYP6P3*, and *CYP6Z1* in *Anopheles coluzzii* in Côte d’Ivoire. High allelic frequencies of knock-down resistance (*kdr*) *L1014F* mutation (range: 0.46–1), relatively low frequencies of the *ace-1R* mutation in the acetylcholinesterase gene associated with target-site insensitivity to carbamates and organophosphates (< 0.5), and elevated activity of insecticide detoxifying enzymes (mainly mixed function oxidases (MFOs), esterase and glutathione S-transferase (GST)), have been reported in Côte d’Ivoire [5, 7, 10]. Recent laboratory (WHO tube and CDC bottle) bioassays against local pyrethroid-resistant *An. gambiae* from Côte d’Ivoire have shown that pyrethroids induce low mortality, whilst pre-exposure to the synergist piperonyl butoxide (PBO) increases the mortality but does not restore fully the susceptibility due to resistance [9]. However, the pyrrole insecticide chlorfenapyr induces higher mortality in resistant *An. gambiae* compared with pyrethroids alone or combined with PBO in Côte d’Ivoire [9].

The present good laboratory practice (GLP) study evaluated the bio-efficacy of a new candidate LLIN, PermaNet^®^ Dual, against pyrethroid-resistant *An. gambiae s.l.* in comparison with the WHO Prequalification Unit, Vector Control Product Assessment Team (PQT/VCP) listed standard LLINs, PermaNet^®^ 2.0 and PermaNet^®^ 3.0, through the conduct of an experimental hut trial and laboratory bioassays in Tiassalé, Côte d’Ivoire. PermaNet^®^ Dual has a mixture of the pyrrole chlorfenapyr and the pyrethroid deltamethrin coated onto a polyester fabric. PermaNet^®^ 3.0 is treated with deltamethrin and PBO, and PermaNet^®^ 2.0 is treated with deltamethrin only. Chlorfenapyr is a pro-insecticide activated by the oxygenase function of cytochrome P450s and oxidative removal of the N-ethoxymethyl group leads to a toxic form identified as CL 303,268. The toxic form uncouples oxidative phosphorylation in the mitochondria, resulting in disruption of the production of adenosine triphosphate and loss of energy, leading to cell dysfunction and ultimately death of the insect [12]. The WHO has received some resistance monitoring data for chlorfenapyr, but these data are insufficient to assess the potential presence of resistance to this insecticide [1]. Deltamethrin is a neurotoxic insecticide and has excito-repellent effects on mosquitoes. PBO, used on PermaNet^®^ 3.0, is a synergist which inhibits mixed function oxidases, blocking the detoxification of pyrethroids and at least partially restoring pyrethroid susceptibility [1, 13]. Pyrethroid-resistant strains of *Anopheles* have so far been found to be susceptible to chlorfenapyr [9, 14–18]. Thus, the hypothesis of the current study was that chlorfenapyr would kill the pyrethroid-resistant *An. gambiae* population in Tiassalé, and PermaNet^®^ Dual would induce higher mortality compared to both PermaNet^®^ 3.0 and PermaNet^®^ 2.0 in the experimental huts, and the supplementary laboratory cone and tunnel bioassays could predict these hut trial outcomes.

## Methods

### Experimental huts and mosquitoes

This semi-field GLP study was conducted at our experimental hut station in the health district of Tiassalé (5° 54′ N, 4° 50′ W), in southern Côte d’Ivoire (West Africa), from March to August 2020. The study was conducted in conformance with the study protocol and with the associated standard operating procedures (SOPs), which helped to ensure the quality and reproducibility of the data generated.

Eight standard West Africa-style experimental huts were refurbished and used for the trial. The experimental huts are located near a large, irrigated rice field where *An. gambiae* mosquitoes are highly abundant and multi-resistant to insecticides, including pyrethroids, carbamates, organophosphates, and organochlorines [19–22]. The *An. gambiae s.l.* population around the Tiassalé experimental huts is only composed of *An. coluzzii*. This species is known to have a strong resistance to pyrethroids in Tiassalé [19–22]. The frequencies of *kdr* and *ace-1*^*R*^ are 0.83 and 0.44, respectively [22].

### Net treatments and treatment arms

This study included the PermaNet^®^ Dual (candidate net), PermaNet^®^ 3.0 and PermaNet^®^ 2.0 (reference nets) and untreated net (negative control). All mosquito nets were new and unwashed nets supplied by Vestergaard Sàrl (Lausanne, Switzerland). The untreated nets were made of polyester fabric without any insecticide. PermaNet^®^ Dual was made of polyester fabric coated with chlorfenapyr at 5.0 g/kg ± 25% and deltamethrin at 2.1 g/kg ± 25%. The unwashed PermaNet^®^ Dual arm was replicated using nets from two different production batches (referred to as A and B). PermaNet^®^ 3.0 was made of polyester fabric coated with 2.1 g/kg ± 25% of deltamethrin on the sides, and polyethylene incorporated with 4.0 g/kg ± 25% of deltamethrin and 25.0 g/kg ± 25% of PBO on the roof. PermaNet^®^ 2.0 was made of polyester fabric coated with 1.4 g/kg ± 25% of deltamethrin.

Eight treatment arms were compared in the experimental huts and laboratory: PermaNet^®^ Dual (A) unwashed; PermaNet^®^ Dual (B) unwashed and washed 20 times; PermaNet^®^ 3.0 unwashed and washed 20 times; PermaNet^®^ 2.0 unwashed and washed 20 times; and untreated control net. The nets were prepared and washed using a soap called “Savon de Marseille” and according to the WHO guidelines on small-scale field hut trials and laboratory testing [23]. The interval of time between two consecutive washes was one day, corresponding to the regeneration time of all the nets. The regeneration time of the PermaNet^®^ Dual (i.e. 1 day) was supplied by Vestergaard. The nets were washed 20 times. Before testing them in the experimental huts, all the unwashed, washed and untreated control nets were deliberately holed with six holes of 4 cm in diameter to stimulate the conditions of torn nets according to the WHO guidelines [23].

### Hut trial procedure

Nets were rotated between the huts following each round of eight consecutive collection days.

One net was hung in each hut, with one net per treatment arm deployed concurrently in the eight huts. Eight replicate nets were used per treatment arm, and each of the eight nets was tested one night per round. The volunteer human sleepers were rotated each night between the huts. The treatment and sleeper rotations were done using a randomized Latin square design to adjust for any variation in mosquito hut entry and attractiveness of volunteers to mosquitoes, respectively. Between the rotations of treatments, the huts were carefully cleaned and aired for one day to minimize the risk of cross-contamination between treatments. The hut trial lasted for 64 days to ensure complete rotation through the huts. In this study, the 64-day trial was long enough to obtain the numbers of mosquitoes needed for statistical analysis. The simulations (> 1000 simulations) targeted a power of > 80% sensitivity. An 8-by-8-Latin square design was considered, targeting an average of 8 mosquitoes per hut per day, with an inter-observational variance of 0.45 for daily observation of mosquito collected. This was powered at a sensitivity of 81.4% to detect a non-inferiority of PermaNet^®^ Dual to the positive control (PermaNet^®^ 3.0 or PermaNet^®^ 2.0), estimating that both will induce 50% mortality at the lowest confidence interval of > 0.7. The volunteer sleepers were asked to note, and were interviewed regarding, any perceived adverse side effects during and at the end of the hut trial.

Each morning, the sleepers collected all of the mosquitoes dead and alive inside the huts and nets, and veranda or exit traps using hand aspirators. The mosquitoes were put in clean plastic cups by collection location, transported to the laboratory. In the laboratory, the mosquitoes were sorted by status (alive or dead; blood fed or unfed), and identified morphologically to genus and species level using taxonomic keys [24, 25]. Live mosquitoes were placed into small clean cups, provided with access to 10% sugar solution via a small cotton wool and held for a 72-h period to record delayed mortality at 24, 48 and 72 h.

### Outcomes measures

The following entomological outcomes were used to evaluate the efficacy of each treatment arm in the current experimental hut trial [23]:

1. Deterrence: proportional reduction in the number of mosquitoes caught in treated hut relative to the number caught in the control hut.
2. Exiting rate: percentage of the mosquitoes collected from the veranda trap out of all mosquitoes collected.
3. Induced exophily: proportional reduction of mosquitoes found in the exit and veranda traps relative to control hut.
4. Blood-feeding: percentage of blood-fed mosquitoes relative to the total collected.
5. Blood-feeding inhibition: proportional reduction in blood feeding percentage in treated huts relative to the control hut.
6. Mortality: percentage of dead mosquitoes found dead in hut in the morning (immediate mortality) or after being caught alive and dead during holding (delayed mortality) in treatment huts out of mosquitoes collected, and corrected for control mortality.
7. Personal protection: the proportional reduction in the number of blood-fed mosquitoes in the treated huts relative to the number of blood-fed mosquitoes in the untreated control.
8. Killing effect: the proportional reduction in the number of mosquitoes killed in the treated huts relative to the number of mosquitoes killed in the untreated control.

The formulas of key entomological outcomes measured in this study are [23]:

$$\mathrm{Deterrence }(\mathrm{\%})=\frac{\mathrm{Nc}-\mathrm{Nt}}{\mathrm{Nc}}\times 100=\left(1-\frac{\mathrm{Nt}}{\mathrm{Nc}}\right)\times 100,$$

where Nt the total number of mosquitoes collected in the treatment hut and veranda/exit traps and Nc the total number of mosquitoes in the control hut and veranda/exit traps.

$$\mathrm{Exiting\,rate }(\mathrm{\%})=\frac{\mathrm{n}}{\mathrm{N}}\times 100,$$

where n is the number of mosquitoes from veranda and window traps, while N is the total number of mosquitoes collected in the hut.

$$\mathrm{Induced\,exophily }(\mathrm{\%})=\frac{\mathrm{Pt}-\mathrm{Pc}}{\mathrm{Pc}}\times 100=\left(\frac{\mathrm{Pt}}{\mathrm{Pc}}-1\right)\times 100,$$

where Pt is the proportion of mosquitoes from veranda and window traps of treated hut while Pc is the number of mosquitoes from veranda and window traps of untreated control hut.

$$\mathrm{Blood}-\mathrm{feeding\,inhibition }\left(\mathrm{\%}\right)=\frac{\mathrm{Pc}-\mathrm{Pt}}{\mathrm{Pc}}\times 100,$$

where Pt the proportion of blood-fed mosquitoes in the treatment hut, and Pc the proportion of blood-fed mosquitoes in the control hut.

$$\mathrm{Personal\,protection }(\mathrm{\%})=\frac{\mathrm{Bc}-\mathrm{Bt}}{\mathrm{Bc}}\times 100=\left(1-\frac{\mathrm{Bt}}{\mathrm{Bc}}\right)\times 100,$$

where Bt the number of blood-fed mosquitoes in the treatment hut and Bc the number of blood-fed mosquitoes in the control hut.

$$\mathrm{Killing\,effect }(\mathrm{\%})=\frac{\mathrm{Kt}-\mathrm{Kc}}{\mathrm{Tc}}\times 100,$$

where Kt is the number of mosquitoes killed in the treatment huts, Kc is the number of mosquitoes killed in the control huts, and Tc is the total number of mosquitoes collected from the control.

### Supporting laboratory testing of net samples

Standard WHO cone bioassays and tunnel tests were conducted with unwashed and washed nets under laboratory conditions, to predict their responses in the field experimental huts. The insecticide susceptible Kisumu strains and field-collected F_0_ generation of pyrethroid-resistant Tiassalé strain of *An. gambiae* were tested with samples (25 cm × 25 cm) cut from each of the eight net types (unwashed and washed net samples) before and after their inclusion into the hut trial [23]. For the tunnel tests, tunnels were divided into two sections by netting samples held in a frame. Nine holes of 1 cm in diameter were cut in each net sample (with one hole located at the centre of the square, and the other eight equidistant and located 5 cm from the border), and the surface of netting available to the mosquitoes was 400 cm^2^ (20 cm × 20 cm).

For each type of net and wash status, 100 non-blood-fed, 2–5-day-old females per strain were subjected to 3-min exposure in cone bioassays in replicates of five mosquitoes per cone according to the WHO guidelines [22]. For the tunnel tests, 100 non-blood-fed, 5–8-day-old mosquito females per strain were subjected to a 15-h exposure overnight using guinea pig as host, following the WHO guidelines [23]. Testing and holding conditions were 27 ± 2 °C and 80 ± 10% relative humidity. The 60-min knockdown for cone bioassays, blood-feeding status for tunnel tests and 24, 48 and 72-h mortality for both methods were recorded.

### Chemical analysis

Pieces of netting were sampled from unwashed and washed PermaNet^®^ Dual (A and B), PermaNet^®^ 3.0 and PermaNet^®^ 2.0 LLINs before and after being used in the hut trial, in accordance with the WHO protocol for chemical analysis [25]. All sampled net pieces were labelled and stored individually in aluminium foil at 3.7–4.2 °C. The net pieces were then shipped to the Vestergaard’s ISO IEC17025 laboratory in Vietnam for chemical analysis of chlorfenapyr and deltamethrin. Deltamethrin and PBO contents were determined following the Collaborative International Pesticides Analytical Council Ltd (CIPAC) (i.e. CIPAC 33/LN/(M2)/3) methods [26]. Briefly, chlorfenapyr content was analysed using in-house method VCL-098-20 that was undergoing CIPAC validation and was still to be published. Chlorfenapyr content was extracted from the PermaNet^®^ Dual net using a mixture of n-hexane and 1,4-dioxane (95:5, v:v) solvents with an internal standard of dibutylphthalate added. The mixture was shaken by shaking machine to extract chlorfenapyr. Extracted solution was filtered through 0.45 μm Teflon membrane and analysed by a normal phase high performance liquid chromatography for chlorfenapyr concentration, detector UV 236 nm.

### Data analysis

Data from the experimental hut trial, cone bioassays and tunnel tests were double entered into Excel and graphs to show mortality and knockdown were then generated. The number of mosquitoes entering each hut was compared among the different treatment arms by negative binomial regression. Proportional outcomes (i.e. deterrence, blood-feeding, exiting or induced exophily and mortality) related to each experimental hut treatment were assessed using generalized linear mixed models (GLMM) using repeated measures approach, which provide a framework for regression modelling of non-normal outcome data. The models were adjusted for the random effects of the volunteer sleepers and experimental huts, and clustering effects by each hut-night of collection. The geometric means were reported due to significant deviations of data from normality (Shapiro–Wilk test for data normality was significant). A significance level of 5% was set for statistical testing. All statistical analyses were conducted using Stata version 16.0 (Stata Corporation; College Station, TX, United States of America).

### Ethical considerations

This study protocol received approval from the national ethics committee of Côte d’Ivoire (reference: N/Ref: 037-20/MSHP/CNESVS-km). All the volunteer sleepers were recruited from the village of Tiassalékro near to our Tiassalé experimental huts after obtaining informed written consent. They were provided with chemoprophylaxis during the trial. A medical doctor was on hand to respond to any side effects of the LLINs and treat any cases of fever.

## Results

### Experimental hut trial

**Fig. 1:**
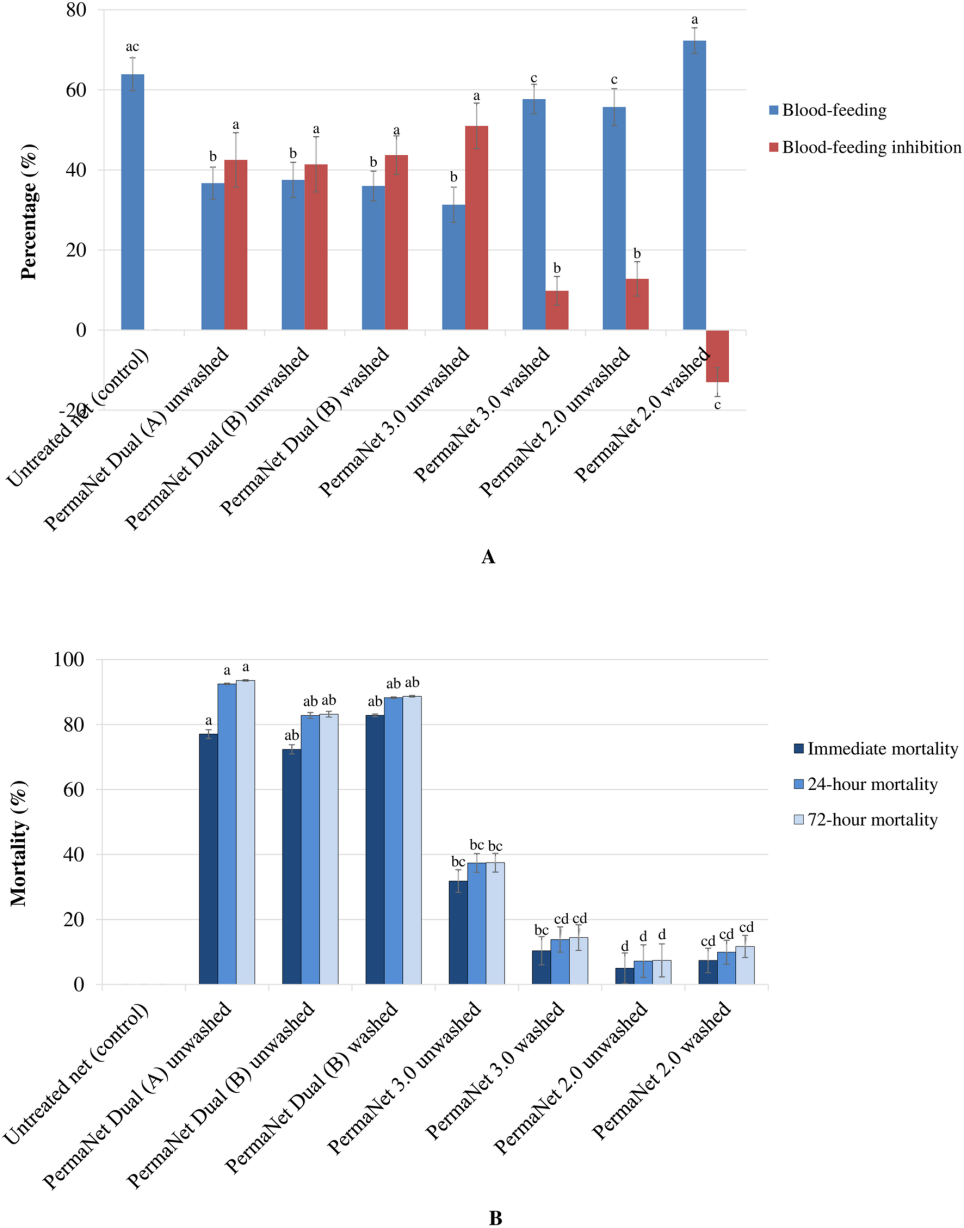
Effects of treatments on blood-feeding (**A**) and immediate and extended mortality (**B**) rates in multiple insecticide-resistant *Anopheles gambiae* from experimental huts in Tiassalé, Côte d’Ivoire. Error bars show the standard error of the mean. Letters indicate the results of the generalized linear mixed model (GLMM). Groups that do not share the same letter for the same post-exposure time (i.e., immediate, 24 h, 72 h) are significantly different

The outcomes of the current experimental hut trial showed that the parameters of the net efficacy against the pyrethroid-resistant *An. gambiae s.l.* Tiassalé population varied as a function of net type and wash status, with the performance of PermaNet^®^ Dual being better overall compared with PermaNet^®^ 3.0 and PermaNet^®^ 2.0 (Fig. 1 and Table 1).

**Table 1:** The outcomes of experimental hut trials of long-lasting insecticidal nets against pyrethroid-resistant *Anopheles gambiae* s.l. in Tiassalé, southern Côte d'Ivoire

Parameter	Summary data	Untreated net (control)	PermaNet® Dual (A) unwashed	PermaNet® Dual (B) unwashed	PermaNet® Dual (B) washed	PermaNet® 3.0 unwashed	PermaNet® 3.0 washed	PermaNet® 2.0 unwashed	PermaNet® 2.0 washed
Deterrence	Total number of females caught	519	421	381	612	326	558	381	669
	Females caught/night: mean ± SEM	8.1 ± 1.0^a,b^	6.6 ± 1.0^b,c^	6.0 ± 0.7^b,c^	9.6 ± 1.5^a,b^	5.1 ± 0.7^c^	8.7 ± 0.9^a,b^	6.0 ± 0.8^b,c^	10.5 ± 1.0^a^
	Deterrence (%)	–	18.9 ± 2.3^a^	26.6 ± 1.9^a^	− 17.9 ± 2.0^b^	37.2 ± 3.3^a^	− 7.5 ± 0.8^b^	26.6 ± 3.0^a^	− 28.9 ± 2.3^c^
Exophily	Number of females in exit traps and veranda	177	295	281	376	238	301	186	326
	Exophily rate (%): mean ± SEM	42.1 ± 4.5^a^	70.7 ± 3.9^b^	70.4 ± 3.8^b^	63.3 ± 3.5^b^	72.8 ± 4.2^b^	54.0 ± 3.6^b^	51.8 ± 4.2^a^	42.4 ± 3.3^a^
	Induced exophily (%)	–	67.9	67.2	50.5	72.9	28.3	23.2	0.6
Blood-feeding	Number of blood-fed females	337	139	132	269	125	304	240	447
	Percentage of blood-fed females (%): mean ± SEM	63.9 ± 4.1^a,c^	36.7 ± 4.0^b^	37.5 ± 4.4^b^	36.0 ± 3.7^b^	31.3 ± 4.4^b^	57.7 ± 3.7^c^	55.7 ± 4.6^c^	72.3 ± 3.2^a^
	Blood-feeding inhibition (%): mean ± SEM	–	42.5 ± 6.8^a^	41.4 ± 6.9^a^	43.7 ± 4.8^a^	51.0 ± 5.7^a^	9.8 ± 3.6^b^	12.8 ± 4.3^b^	− 13.0 ± 3.6^c^
Mortality Immediate mortality	Number of dead females morning (immediate mortality)	1	372	338	509	153	157	56	133
	Immediate mortality (%): mean ± SEM	0.0 ± 0.4^d^	77.1 ± 1.3^a^	72.4 ± 1.4^a,b^	82.8 ± 0.4^a,b^	31.9 ± 3.5^b,c^	10.6 ± 4.3^ c,d^	5.0 ± 4.7^d^	7.4 ± 3.8^ c,d^
	Immediate mortality corrected for control (%): mean ± SEM	0	77.1 ± 1.3^a^	72.4 ± 1.4^a,b^	82.8 ± 0.4^a,b^	31.8 ± 3.5^b,c^	10.4 ± 4.3^ c,d^	5.0 ± 4.7^d^	7.4 ± 3.8^ c,d^
24-h mortality	Number of dead females after 24 h (delayed mortality)	1	14	11	33	10	22	13	24
	Total number of dead females after 24 h	2	386	349	542	163	179	69	157
	24-h mortality (%): mean ± SEM	0.1 ± 0.6^d^	92.5 ± 0.2^a^	82.9 ± 0.9^a,b^	88.2 ± 0.2^a,b,c^	37.4 ± 2.9^b,c^	14.2 ± 3.9^c,d^	7.2 ± 5.0^d^	10.3 ± 3.6^c,d^
	24-h mortality corrected for control (%): mean ± SEM	–	92.5 ± 0.2^a^	82.8 ± 0.9^a,b,c^	88.2 ± 0.2^a,b,c^	37.8 ± 2.9^b,c^	13.8 ± 3.9^c,d^	7.2 ± 5.0^d^	9.9 ± 3.7^c,d^
72-h mortality	Number of dead females after 72 h (delayed mortality)	2	20	13	35	11	32	16	34
	Total number of dead females after 72 h	3	392	351	544	164	189	72	167
	72-h mortality (%): mean ± SEM	0.1 ± 0.7^d^	93.6 ± 0.2^a^	83.3 ± 0.9^a,b^	88.7 ± 0.2^a,b^	37.5 ± 2.9^b,c^	14.8 ± 3.9^c,d^	7.4 ± 5.1^d^	11.9 ± 3.4^c,d^
	72-h mortality corrected for control (%): mean ± SEM	–	93.6 ± 0.2^a^	83.2 ± 0.9^a,b^	87.7 ± 0.2^a,b^	37.5 ± 2.9^b,c^	14.4 ± 3.9^c,d^	7.4 ± 5.1^d^	11.7 ± 3.4^c,d^
Personal protection	Personal protection (%)	–	58.8 ± 3.5^a^	60.8 ± 3.8^a^	20.2 ± 1.1^b^	62.9 ± 4.2^a^	9.8 ± 0.7^c^	28.8 ± 1.9^b^	− 32.6 ± 2.2^d^
Killing effect (%)	Killing effect (%)	–	74.8 ± 4.2^a^	66.9 ± 4.3^a^	104.0 ± 6.3^a^	30.8 ± 2.2^b^	35.6 ± 1.9^b^	13.1 ± 0.9^c^	31.4 ± 1.6^b^

Each washed net sample was washed 20 times. The killing effect was calculated for the 72-h mortality. The percentages represent the geometric means of outcomes. Letters indicate the results of the generalized linear mixed model (GLMM). Groups that do not share the same letter for the same post-exposure time (i.e., immediate, 24 h, 72 h) are significantly different

% percentage, *h* hour, *SEM* standard error of the mean

### Mosquito hut entry and deterrence

A total of 3867 female *An. gambiae* were collected in the huts. Hut entry rate (geometric mean ± standard error of the mean) varied between 5.1 ± 0.7 females/hut/night in washed PermaNet^®^ 3.0 and 10.5 ± 1.0 females/hut/night in washed PermaNet^®^ 2.0, and differed significantly by between the treatment arms (χ^2^ = 31.35; df = 7; p < 0.05) (Table 1). Before washing, the deterrence with PermaNet^®^ Dual (A) (18.9 ± 2.3%) was statistically comparable to PermaNet^®^ Dual (B) (26.6 ± 1.9%) (z = − 0.57; p = 0.567), PermaNet^®^ 3.0 (37.2 ± 3.3%) (z = − 1.46; p = 0.145) and PermaNet^®^ 2.0 (26.6 ± 3.0%) (z = − 0.57; p = 0.567). Similarly, the deterrence in unwashed PermaNet^®^ Dual (B) did not differ significantly compared with unwashed PermaNet^®^ 3.0 (z = − 0.88; p = 0.376) and unwashed PermaNet^®^ 2.0 (z = − 0.02; p = 0.988). After washing, PermaNet^®^ Dual (B) deterrence declined significantly from 26.6 ± 1.9% to − 17.9 ± 2.0% (z = − 2.75; p = 0.006). However, the deterrence in washed PermaNet^®^ Dual (B) did not differ significantly from washed PermaNet^®^ 2.0 (− 28.9 ± 2.3%) (z = − 0.54; p = 0.586) and washed PermaNet^®^ 3.0 (− 7.5 ± 0.8%) (z = − 0.53; p = 0.598).

### Exiting effect and induced exophily

Before washing, exiting effect of PermaNet^®^ Dual (A) (70.7 ± 3.9%) was statistically similar compared with PermaNet^®^ Dual (B) (70.4 ± 3.8%), (z = 1.20; p = 0.230), PermaNet^®^ 3.0 (72.8 ± 4.2%) (z = 1.86; p = 0.063) and PermaNet^®^ 2.0 (51.8 ± 4.2%) (z = − 0.72; p = 0.470) (Table 1). The existing effect of unwashed PermaNet^®^ Dual (B) was not significantly different to unwashed PermaNet^®^ 3.0 (z = 0.50; p = 0.621) and unwashed PermaNet^®^ 2.0 (z = − 1.82; p = 0.068). Washing did not significantly alter the exiting effect in PermaNet^®^ Dual (B), as there was no statistical difference between unwashed and washed samples (z = − 0.35; p = 0.728). Washed PermaNet^®^ Dual (B) (63.3 ± 3.5%) had significantly higher exiting effect than that induced by washed PermaNet^®^ 2.0 (42.4 ± 3.3%) (z = − 2.11; p = 0.035) and the untreated control net (42.1 ± 4.5%) (z = − 2.07; p = 0.038), but statistically similar to washed PermaNet^®^ 3.0 (54.0 ± 3.6%) (z = − 0.81; p = 0.417). No significant differences were found in the induced exophily between any of the net types (all p > 0.05).

### Blood-feeding and personal protection

The blood-feeding effect of the nets in wild pyrethroid-resistant *An. gambiae* that entered the trial huts and personal protection are presented in Fig. 1A and Table 1. Before washing, the percentage blood-feeding with PermaNet^®^ Dual (A) (36.7 ± 4.0%) did not differ significantly from PermaNet^®^ Dual (B) (37.5 ± 4.4%) and PermaNet^®^ 2.0 (55.7 ± 3.7%), but was significantly higher than PermaNet^®^ 2.0 (31.3 ± 4.4%) (z = − 2.17; p = 0.030). The percentage blood-feeding for unwashed PermaNet^®^ Dual (B) was similar to unwashed samples of PermaNet^®^ 2.0 (z = 1.74; p = 0.081) and PermaNet® 3.0 (z = − 1.49; p = 0.136). Washing 20 times did not influence significantly the percentage blood-feeding in PermaNet^®^ Dual (B) (z = 0.10; p = 0.921). After washing, the percentage blood-feeding of PermaNet^®^ Dual (B) (36.0 ± 3.7%) was substantially lower, but without significant differences when compared with PermaNet^®^ 3.0 (57.7 ± 3.7%) (z = 1.71; p = 0.087), PermaNet^®^ 2.0 (72.3 ± 3.2%) (z = 1.78; p = 0.076) and the untreated control net (z = 1.72; p = 0.085). For blood-feeding inhibition, unwashed PermaNet^®^ Dual (A) (42.5 ± 6.8%) was similar to unwashed PermaNet^®^ Dual (B) (41.4 ± 6.9%) (z = 0.25; p = 0.802) and washed PermaNet^®^ Dual (B) (43.7 ± 4.8%) that was not affected by washing (z = − 0.41; p = 0.682). Compared with PermaNet^®^ 3.0, the blood-feeding inhibition of PermaNet^®^ Dual (B) was significantly higher before washing (z = − 0.72; p = 0.469), and significantly lower after washing (z = − 1.92; p = 0.045). However, both unwashed and washed PermaNet^®^ Dual (B) provided, respectively, significantly higher blood-feeding inhibition than unwashed and washed PermaNet^®^ 2.0 (all p < 0.05). The personal protection was similar between unwashed samples of PermaNet^®^ Dual (A) (58.8 ± 3.5%) and PermaNet^®^ Dual (B) (60.8 ± 3.8%) (z = − 0.04; p = 0.968). Unwashed PermaNet^®^ Dual (B) resulted in a personal protection that was similar to unwashed PermaNet^®^ 3.0 (62.9 ± 4.2%) (z = 0.21; p = 0.738), and significantly different from unwashed PermaNet^®^ 2.0 (28.8 ± 1.9%) (z = 3.21; p = 0.001). With 20 washes, the personal protection with PermaNet^®^ Dual (B) declined significantly from 60.8 ± 3.8% to 20.2 ± 1.1% (z = − 2.96; p = 0.003). However, washed PermaNet^®^ Dual (B) provided significantly higher personal protection compared with their washed counterparts of PermaNet^®^ 3.0 (9.8 ± 0.7%) (z = 2.89; p = 0.004) and PermaNet^®^ 2.0 (− 32.6 ± 2.2%) (z = 2.93; p = 0.003).

### Mortality and killing effect

The percentage mortality (immediate mortality and delayed mortality) and killing effect in free-flying pyrethroid-resistant *An. gambiae* that entered the experimental huts are indicated in Fig. 1B, with further details provided in Table 1. The mortality increased with the time of assessment in both unwashed and washed samples of all types of nets, but without significant differences from 24 to 72-h post-collection within each of net types (all p > 0.05). Before washing, the mortality with PermaNet^®^ Dual (A) (93.6 ± 0.2%) was significantly higher compared with PermaNet^®^ Dual (B) (83.2 ± 0.9%) (z = − 2.07; p = 0.038), PermaNet^®^ 3.0 (37.5 ± 2.9%) (z = − 1.98; p = 0.047) and PermaNet^®^ 2.0 (7.4 ± 5.1%) (z = − 5.60; p = 0.001). Unwashed PermaNet® Dual (B) induced significantly higher mortality than that caused by unwashed PermaNet^®^ 2.0 (z = − 3.22; p = 0.001), but similar when compared with unwashed PermaNet^®^ 3.0 (z = 0.69; p = 0.493). After 20 washes, the mortality with PermaNet^®^ Dual (B) increased from 83.2 ± 0.9% to 87.7 ± 0.2%, without significant difference between unwashed and washed samples (z = − 1.01; p = 0.313). However, the mortality with washed PermaNet^®^ Dual (B) (87.7 ± 0.2%) was significantly higher than washed PermaNet 3.0 (14.4 ± 3.9%) (z = − 3.33; p = 0.001) and washed PermaNet 2.0 (11.7 ± 3.4%) (z = − 2.96; p = 0.003). Additional file summarizes the mortality of pyrethroid-resistant *An. gambiae* Tiassalé population in more detail (see Additional file 1: Table S1). The killing effect was higher with washed PermaNet^®^ Dual (B) (104.0 ± 6.3%), followed by unwashed PermaNet^®^ Dual (A) (74.0 ± 4.2%) and unwashed PermaNet^®^ Dual (B) (66.9 ± 4.3%). PermaNet^®^ Dual (B) (66.9 ± 4.3% and 104.0 ± 6.3%) had significantly higher killing effect than PermaNet^®^ 3.0 (30.8 ± 2.2% and 35.6 ± 1.9%) and PermaNet^®^ 2.0 (13.1 ± 0.9% and 31.4 ± 1.6%), when unwashed or washed (all p < 0.05).

### Perceived side effects

No negative side effects or complaints were reported among the eight hut sleepers who participated in the interviews.

### Supporting laboratory bioassays

#### Cone bioassay

**Fig. 2:**
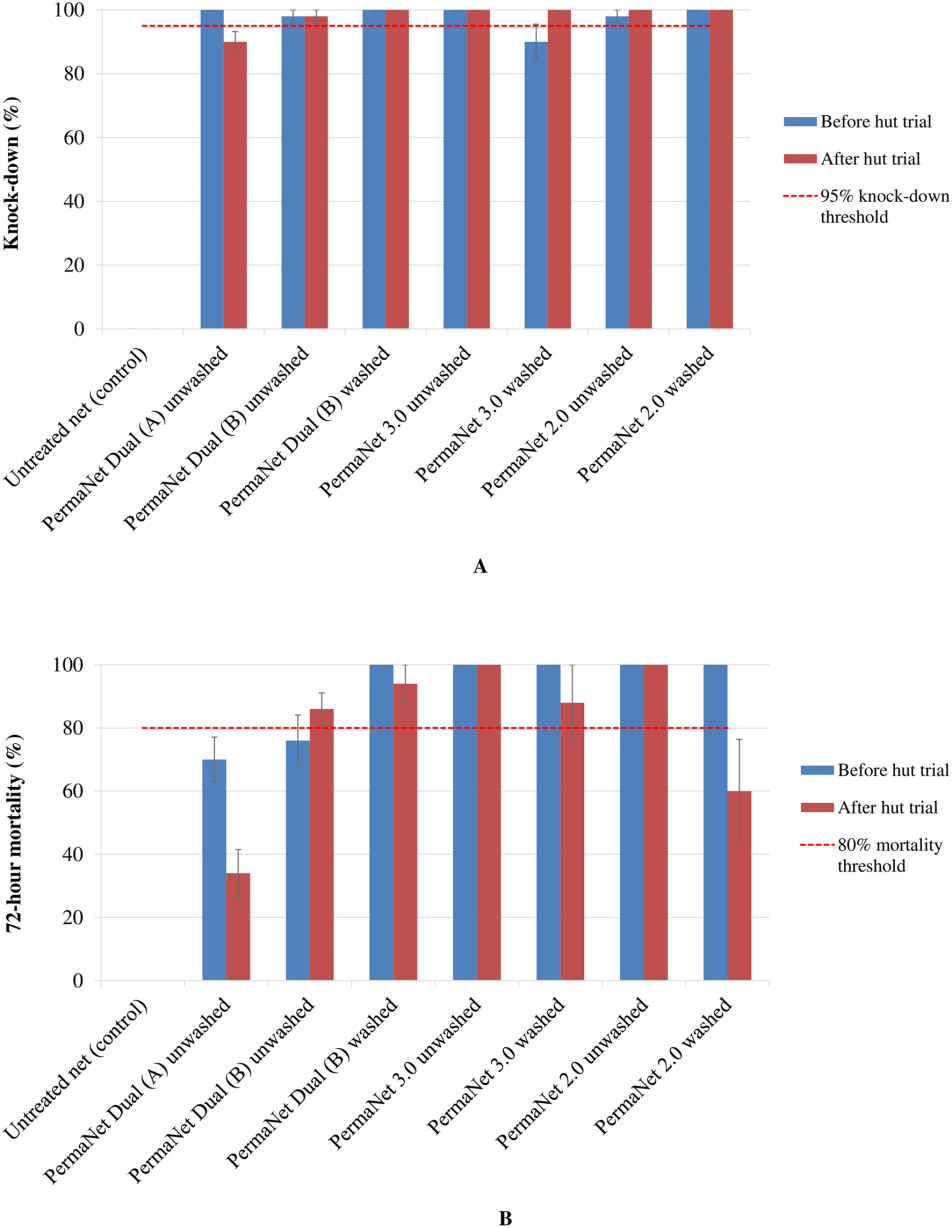
Mean knock-down (**A**) and corrected mortality (**B**) rates in the susceptible *Anopheles gambiae s.s.* Kisumu strain exposed to net samples using cone bioassays before and after the experimental hut trial in Tiassalé, Côte d’Ivoire. KD60: Knock-down at 60 min. Error bars show the standard error of the mean

**Fig. 3:**
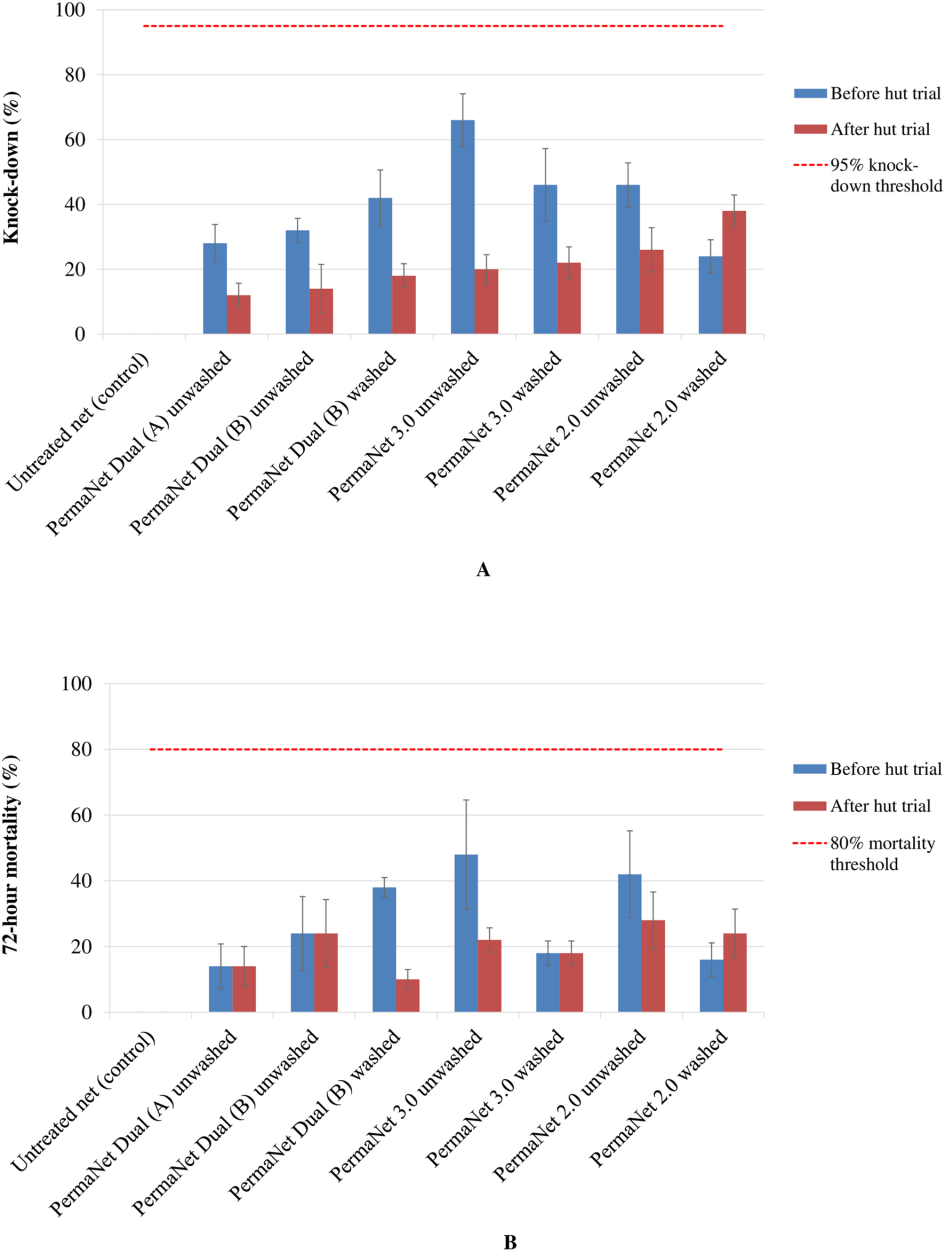
Mean knock-down (**A**) and corrected mortality (**B**) rates in the pyrethroid-resistant *Anopheles gambiae s.l.* Tiassalé strain exposed to net samples using cone bioassays before and after the experimental hut trial in Tiassalé, Côte d’Ivoire. KD60: Knock-down at 60 min. Error bars show the standard error of the mean

For the susceptible *An. gambiae *sensu stricto (*s.s*.) Kisumu strain, the knock-down was high in all the nets. The knock-down ranged from 90.0% to 100.0%, with PermaNet^®^ Dual from 90.0% to 100.0% with PermaNet^®^ 3.0 and from 98.0% to 100.0% with PermaNet^®^ 2.0, with percentages being higher than the WHO 95% knock-down threshold before and after washing or the hut trial (Fig. 2A). Compared to the WHO 80% mortality threshold, the mortality was lower for unwashed PermaNet^®^ Dual (A) (70.0% for unused and 34.0% for used samples) and unwashed PermaNet^®^ Dual (B) (76.0% for unused samples), but higher for unwashed PermaNet® Dual (B) (86.0% for used samples) and washed PermaNet^®^ Dual (B) (100.0% for unused and 94.0% for used samples) (Fig. 2B). The mortality with unwashed or washed and unused or used samples of PermaNet^®^ 3.0 and PermaNet® 2.0 was higher than 80%, excepted for used samples of washed PermaNet^®^ 2.0 (Fig. 2B). Additional file shows the susceptible *An. gambiae s.s.* Kisumu strain mortality with cone bioassays more detail (see Additional file 2: Table S2). Thus, PermaNet^®^ Dual (A and B), PermaNet^®^ 3.0 and PermaNet^®^ 2.0 all passed the WHO cone bioassay bio-efficacy criteria (≥ 95% knockdown or ≥ 80% mortality [23]) (Fig. 2). With the pyrethroid-resistant *An. gambiae s.l.* Tiassalé strain, knock-down and mortality were very low, varied respectively from 12.0% to 66.0% and from 10.0% to 48.0%), and were thus lower than the 95% knock-down or 80% mortality thresholds across all nets (i.e. PermaNet® Dual, PermaNet^®^ 3.0 and PermaNet^®^ 2.0), whether unwashed or washed, and unused or used (Fig. 3). Additional file shows the pyrethroid-resistant *An. gambiae s.l.* Tiassalé strain mortality with cone bioassays more detail (see Additional file 3: Table S3).

#### Tunnel test

**Fig. 4:**
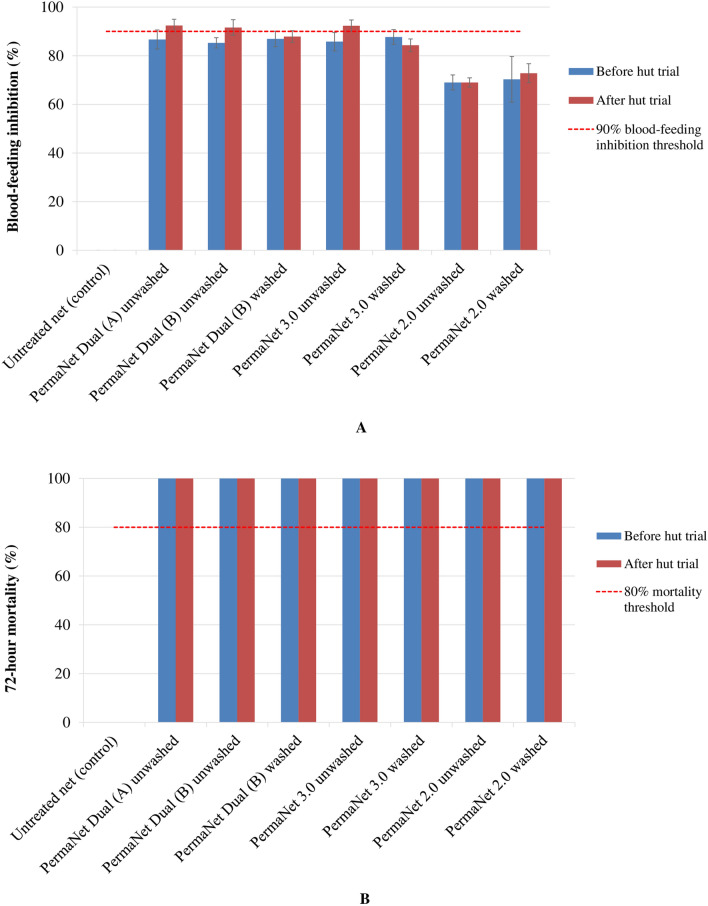
Mean blood-feeding inhibition (**A**) and corrected mortality (**B**) rates in the susceptible *Anopheles gambiae* s.s. Kisumu strain exposed to net samples using tunnel tests before and after experimental hut trial in Tiassalé, Côte d’Ivoire. Error bars show the standard error of the mean

**Fig. 5:**
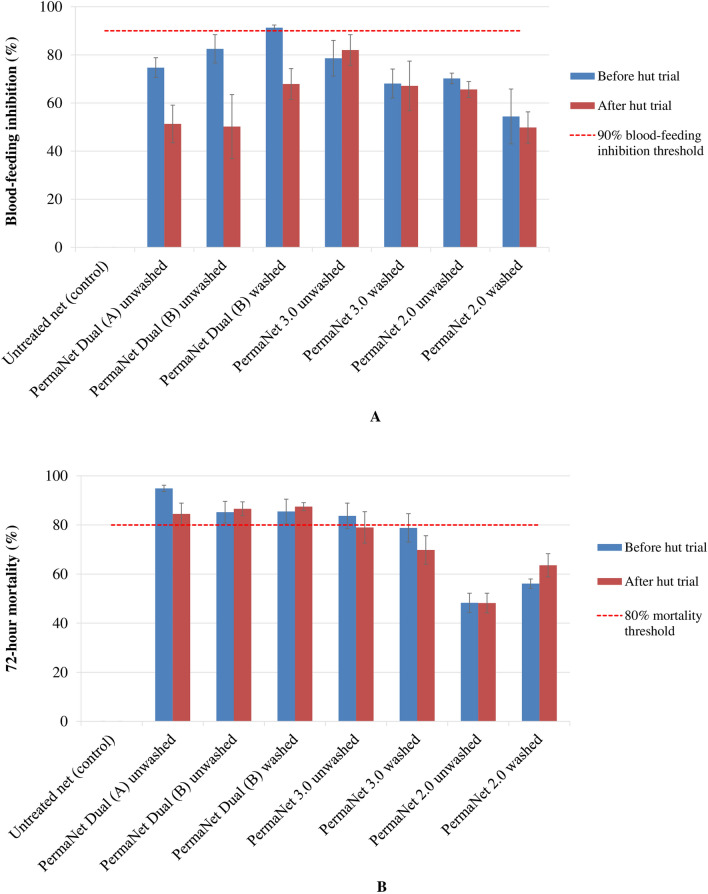
Mean of blood-feeding inhibition (**A**) and corrected mortality (**B**) rates in the pyrethroid-resistant *Anopheles gambiae s.l.* Tiassalé strain exposed to net samples using tunnel tests before and after experimental hut trial in Tiassalé, Côte d’Ivoire. Error bars show the standard error of the mean

The tunnel test outcomes confirmed the cone bioassay results for the susceptible *An. gambiae s.s.* Kisumu strain, with strong blood-feeding inhibition and high mortality in all nets being above the WHO tunnel test thresholds (≥ 90% blood-feeding inhibition or ≥ 80% mortality) (Fig. 4). The blood-feeding inhibition was high only in unwashed and used samples of PermaNet^®^ Dual (A) (92.4%), PermaNet^®^ Dual (B) (91.6%) and PermaNet^®^ 3.0 (92.3%) (Fig. 4A). The mortality was very high (100%) in unwashed or washed and unused or used samples of all nets (PermaNet^®^ Dual, PermaNet^®^ 3.0 and PermaNet^®^ 2.0) (Fig. 4B), thus showing their good efficacy against this pyrethroid-susceptible *An. gambiae* Kisumu strain. Additional file shows the Kisumu strain mortality with tunnel tests in more detail (see Additional file 4: Table S4). For the pyrethroid-resistant *An. gambiae s.l.,* only washed and unused PermaNet^®^ Dual (A) induced a strong blood-feeding inhibition (91.3%) being above the WHO tunnel cut-off of 90% (Fig. 5A). However, the mortality with all samples (unwashed or washed and unused or used) of PermaNet^®^ Dual (85.2–94.9%) were higher compared with PermaNet^®^ 3.0 and PermaNet^®^ 2.0 (Fig. 5B), and consistent with the higher efficacy of PermaNet^®^ Dual against the free-flying pyrethroid-resistant *An. gambiae s.l.* Tiassalé strain populations observed in the concurrent experimental hut trials. Additional file shows the pyrethroid-resistant Tiassalé strain mortality with tunnel tests in more detail (see Additional file 5: Table S5).

### Comparison of laboratory bioassay results with hut trial results

Comparing the laboratory cone bioassay and tunnel test results with the experimental hut trial results with the same pyrethroid-resistant *An. gambiae s.l.* strain showed that these laboratory bioassays predicted the response in the huts for PermaNet^®^ 3.0 and PermaNet^®^ 2.0. Indeed, the unwashed and washed PermaNet^®^ 3.0 and PermaNet^®^ 2.0 induced relatively lower mortality in the pyrethroid-resistant *An. gambiae* strain with the cone bioassays, the tunnel test and the hut trial. Focussing on the bioefficacy of PermaNet^®^ Dual, the respective mortality rates in the cone, tunnel and hut were 14.0%, 84.5% and 94.9% for unwashed PermaNet^®^ Dual (A), 24.0%, 86.5% and 90.2% for unwashed PermaNet^®^ Dual (B), and 10.0%, 87.5% and 90.4% for washed PermaNet^®^ Dual (B). This suggests that laboratory tunnel tests are more predictive of the performance of PermaNet^®^ Dual in experimental huts. Additionally, there were correlations in the blood-feeding rates and blood-feeding inhibition between the tunnel test results and the hut trial results. The high mortality and strong blood-feeding inhibition of PermaNet^®^ Dual in tunnel tests with the pyrethroid-resistant *An. gambiae* Tiassalé strain shows that the chlorfenapyr component of PermaNet^®^ Dual provided good efficacy against pyrethroid-resistant *Anopheles*.

### Chemical contents

Table 2 shows the chemical contents of PermaNet^®^ Dual (A and B), PermaNet^®^ 3.0 and PermaNet^®^ 2.0 before washing, after washing and after testing in the experimental huts. Before washing and testing, the initial contents of deltamethrin of the PermaNet^®^ Dual (batch A: 1.84 g/kg and batch B: 2.41 g/kg), PermaNet® 3.0 (side: 2.10 g/kg and roof: 3.82 g/kg) and PermaNet® 2.0 (1.34 g/kg), chlorfenapyr in the PermaNet^®^ Dual (batch A: 4.25 g/kg and batch B: 4.98 g/kg), and PBO in the PermaNet^®^ 3.0 (roof: 23.00 g/kg) complied with the target specification (i.e. dose interval limits) and, hence, met the tolerance limits required by the WHO [23]. Moreover, the low within sample variation (i.e. low standard errors) in the initial contents of deltamethrin and chlorfenapyr in PermaNet^®^ Dual was low, indicating a good homogeneity of the active ingredients’ distribution over this net. After the completion of the 64-day hut trial, the chemical contents of unwashed PermaNet^®^ Dual, PermaNet^®^ 3.0, and PermaNet^®^ 2.0 remained close to the initial concentrations, suggesting that use of the LLINs in testing did not result in any major changes in the net chemical concentrations. However, in unwashed PermaNet^®^ Dual (A), the contents of chlorfenapyr (24.9%) and deltamethrin (17.4%) were different between unused and used samples, and these differences may be due to the variations in the original loading of insecticides in different samples tested. Washing (20 washes) resulted in substantial reductions of all chemical contents in all types of nets, but with large differences among the active ingredient and net types. Indeed, the deltamethrin content reductions were much higher in washed PermaNet^®^ 2.0 (94.0% and 92.5%) and PermaNet^®^ 3.0 side (89.5% and 91.9%) compared with washed PermaNet^®^ Dual (B) (56.8% and 49.0%) before and after the hut trial, respectively, revealing higher wash retention of deltamethrin in PermaNet^®^ Dual compared with PermaNet^®^ 2.0 and PermaNet^®^ 3.0. After washing, the chlorfenapyr contents in the PermaNet^®^ Dual (B) declined by 63.1% before and 55.0% after the hut trial.

**Table 2:** Active ingredient contents of long-lasting insecticidal net samples used in the experimental hut trial in Tiassalé, Côte d’Ivoire

Net sample	Unit	Dose interval limit	Before hut trial			After hut trial			
		Mean ± SE [95% CI]	Unwashed (initial reference)	Washed		Unwashed		Washed	
				Dose mean	% reduction	Dose mean	% reduction	Dose mean	% reduction
PermaNet® Dual (A)									
Deltamethrin	g/kg	2.1 ± 25% [1.58–2.63]	1.84 ± 0.15	–	–	2.16 ± 0.17	− 17.4	–	–
Chlorfenapyr	g/kg	5.0 ± 25% [3.75–6.25]	4.25 ± 0.50	–	–	5.31 ± 0.57	− 24.9	–	–
Mass per unit area	g/m^2^	40 ± 10% [36–44]	39.80 ± 0.74	–	–	38.78 ± 0.59	2.6	–	–
PermaNet® Dual (B)									
Deltamethrin	g/kg	2.1 ± 25% [1.58—2.63]	2.41 ± 0.27	1.04 ± 0.15*	56.8	2.47 ± 0.20	− 2.5	1.23 ± 0.17*	49.0
Chlorfenapyr	g/kg	5.0 ± 25% [3.75–6.25]	4.98 ± 0.42	1.84 ± 0.26*	63.1	5.01 ± 0.38	− 0.6	2.24 ± 0.31*	55.0
Mass per unit area	g/m^2^	40 ± 10% [36–44]	41.12 ± 0.35	41.94 ± 0.20	− 2.0	41.46 ± 0.23	− 2.0	42.20 ± 0.28	− 2.6
PermaNet® 3.0									
Deltamethrin (side)	g/kg	2.1 ± 25% [1.58–2.63]	2.10	0.22*	89.5	2.09	0.5	0.17*	91.9
Mass per unit area (side)	g/m^2^	40 ± 10% [36–44]	41.60	42.90	− 3.1	41.50	0.2	41.60	0
Deltamethrin (roof)	g/kg	4.0 ± 25% [3.00–5.00]	3.82	3.71	2.9	3.89	− 1.8	3.61	5.5
PBO (roof)	g/kg	25 ± 25% [18.75–31.25]	23.00	16.40*	28.7	19.80	13.9	16.20*	29.6
Mass per unit area (roof)	g/m^2^	30 ± 10% [27–33]	38.60*	44.20*	− 14.5	37.40*	3.1	43.60*	− 14.5
PermaNet® 2.0									
Deltamethrin	g/kg	1.4 ± 25% [1.05 – 1.75]	1.34	0.08*	94.0	1.45	− 8.2	0.10*	92.5
Mass per unit area	g/m^2^	40 ± 10% [36–44]	43.40	43.70	− 0.7	43.40	0.0	43.40	0.0

Mean with asterisk (*) is within the target specification dose interval limits

Mean without asterisk (*) is outside the target specification dose interval limits

*SE* standard error of the mean, *CI* confidence interval

## Discussion

The current study evaluated the efficacy of PermaNet^®^ Dual, a new candidate net coated with a mixture of chlorfenapyr and deltamethrin, against *An. gambiae* in a small-scale GLP hut study in Tiassalé, Côte d’Ivoire, with supporting exploratory laboratory cone and tunnel bioassays. WHO-prequalified PermaNet^®^ 3.0 (co-treated with deltamethrin and PBO) and PermaNet^®^ 2.0 (treated with deltamethrin only) were used as the reference nets. In this GLP hut study, PermaNet^®^ Dual performed better than PermaNet^®^ 3.0 and PermaNet^®^ 2.0, in terms of mortality and blood-feeding inhibition, for both unwashed and washed samples. Unlike the cone bioassays, the tunnel tests successfully confirmed the field efficacy of the nets. Briefly, this hut trial showed the benefit of mixing deltamethrin and chlorfenapyr together in a long-lasting net, PermaNet^®^ Dual, for an effective control of pyrethroid-resistant *Anopheles*.

The current experimental hut study demonstrated that PermaNet^®^ Dual had increased efficacy against highly pyrethroid-resistant *An. gambiae*. Indeed, hut mortality of *An. gambiae* Tiassalé strain was significantly higher with PermaNet^®^ Dual (> 83%) in comparison with PermaNet^®^ 3.0 (< 38%) and PermaNet^®^ 2.0 (< 12%), for both unwashed and 20 times washed samples (Fig. 1B and Table 1). This PermaNet^®^ Dual efficacy was similar to chlorfenapyr and alpha-cypermethrin mixture-coated Intercept G2 in West and East Africa [16, 17, 27–32]. The good performance of PermaNet^®^ Dual might be attributed to the susceptibility of the highly pyrethroid-resistant *An. gambiae* s.l. to chlorfenapyr [9, 11]. Due to the novel mode of action of chlorfenapyr, the present pyrethroid-resistance mechanisms did not provide any cross-resistance to this insecticide [12]. Indeed, chlorfenapyr-treated tool has been shown to control a number of different multiple-insecticide-resistant *Anopheles* populations [14, 15, 27–32].

This hut study showed that the percentage blood-feeding in wild pyrethroid-resistant *An. gambiae* population was lower for PermaNet^®^ Dual (unwashed and washed) compared with PermaNet^®^ 2.0 (unwashed and washed) and washed PermaNet^®^ 3.0, but slightly higher than unwashed PermaNet^®^ 3.0 (Fig. 1A and Table 1). Likewise, blood-feeding inhibition with PermaNet^®^ Dual was comparable or stronger than with PermaNet^®^ 2.0 and washed PermaNet^®^ 3.0. This PermaNet^®^ Dual blood-feeding inhibition outcome is consistent with that caused by Intercept G2 in earlier hut trials in West Africa [27–31], and may be explained by the irritant effects of the deltamethrin [32–35]. While unwashed PermaNet^®^ Dual and PermaNet^®^ 3.0 produced similar levels of blood-feeding inhibition, the 20 times washed PermaNet^®^ Dual induced a statistically greater blood-feeding inhibition in comparison with both PermaNet^®^ 3.0 and PermaNet^®^ 2.0 washed 20 times. This suggests that PermaNet^®^ Dual may have performed better than PermaNet^®^ 3.0 and PermaNet^®^ 2.0 over time. PermaNet^®^ Dual effectiveness for inhibiting blood-feeding could imply its good potential for reducing the human-biting and blood-feeding, adding value into its killing effects in malaria vectors.

In the present hut study, unwashed PermaNet® Dual deterrence effect against the wild pyrethroid-resistant *An. gambiae* population was comparable to that recorded in PermaNet® 2.0 and PermaNet^®^ 3.0. The deltamethrin component of PermaNet^®^ Dual has a deterrence effect and may have induced exiting in mosquitoes. However, the 20 times washed PermaNet^®^ Dual did not have a deterrence effect (Table 1), probably due to a reduction of the chemical contents (Table 2). A dipped chlorfenapyr net did seem to have a deterrence effect, but did not have a significant effect on exiting rates in West Africa [15, 16, 26–31]. The higher deterrence effect may be due to both the chlorfenapyr and deltamethrin components, but higher exiting rate would likely be due only to the deltamethrin component. Thus, PermaNet^®^ Dual may have both deterrent and excito-repellency effects, providing personal protection against pyrethroid-resistant *An. gambiae* mosquitoes, and reducing human-vector contacts, which may, in turn, lead to an increased user acceptance [36].

While standard laboratory cone bioassays failed to predict PermaNet^®^ Dual efficacy against pyrethroid-resistant *An. gambiae s.l*., tunnel tests successfully predicted its efficacy against the same strain in the semi-field experimental huts. Both cone and tunnel bioassays met the WHO criteria [44], with the susceptible *An. gambiae s.s.* Kisumu strain. However, *An. gambiae s.s.* Kisumu strain mortality against unwashed and used PermaNet® Dual (A) was lower (34%) with cone bioassay (Fig. 2B) probably due to the reduction of the chemical bioavailability on netting fibre surface of net samples tested, or the inappropriateness of cone bioassay method for evaluation of the chlorfenapyr-coated PermaNet^®^ Dual as the mortality was higher (100%) with tunnel test (Fig. 4B). With the wild pyrethroid-resistant *An. gambiae s.l.* Tiassalé strain, only the tunnel tests achieved high efficacy that was consistent with that observed in the huts with PermaNet^®^ Dual. The difference in PermaNet^®^ Dual efficacy between both *Anopheles* Kiumu and Tiassalé strains may be explained by the difference in the levels of their resistance to insecticides. However, the lower efficacy of PermaNet^®^ Dual against pyrethroid-resistant *An. gambiae s.l.* with cone bioassays (daytime and 3-min exposure) compared with tunnel tests (night-time and 15-h exposure) may be attributable to the slow mode of action of chlorfenapyr and that mosquitoes need to be metabolically active for the activation of the chlorfenapyr pro-insecticide [12, 13, 37]. Indeed, chlorfenapyr has a previously been observed to have a slower action and delayed toxic activity of 2–3 days post-exposure compared to other insecticides (pyrethroids and organophosphates) used in mosquito vector control [14, 28, 29]. The cone bioassay method was developed to assess the bioefficacy of pyrethroid-only LLINs, and the use of this method to test LLINs containing non-neurotoxic insecticides, such as chlorfenapyr, may not necessarily be predictive of field impact, even with increased exposure time of mosquitoes inside the cones [16, 27]. In contrast, the tunnel test results with pyrethroid-resistant *An. gambiae s.l.* Tiassalé strain (field-collected F_0_ generation) were more predictive of PermaNet^®^ Dual efficacy in huts, as observed for other chlorfenapyr-coated nets, such as Interceptor^®^ G2 [16, 27, 28]. Tunnel tests provide an increased exposure time of mosquitoes to the LLIN samples being tested, and being an overnight exposure. As the female mosquitoes in the tunnel tests are also exhibiting host-seeking behaviour and are, therefore, metabolically active, the activation of chlorfenapyr following tarsal pickup by mosquitoes may be more effective. The tunnel test was a better predictor of PermaNet^®^ Dual field efficacy because exposure occurred at night when host-seeking mosquitoes are more vulnerable to chlorfenapyr. Tunnel test thus is a more reliable technique to assess the efficacy of a chlorfenapyr-treated net prior to field trials against free-flying mosquitoes [16, 38, 39].

In PermaNet^®^ Dual, deltamethrin and chlorfenapyr contents varied slightly, but complied with the dose interval limits of target specification [23]. This revealed a good homogeneity of the active ingredients’ distribution over and a good retention of these active ingredients in PermaNet^®^ Dual, thus resulting in high and similar mortality and blood-feeding inhibition between unwashed samples of PermaNet^®^ Dual (A) and PermaNet^®^ Dual (B). The reduction of chemical contents due to the loss of chlorfenapyr and deltamethrin with 20 washes had no apparent effect on PermaNet^®^ Dual efficacy as the washed samples were still producing high mortality against the pyrethroid-resistant mosquitoes. Overall, PermaNet^®^ Dual chemical bioavailability was sufficient and produced high mortality in pyrethroid-resistant *Anopheles* mosquitoes after 20 standardized washes (corresponding to a 3-year use) and over the hut trial.

The high vector mortality and personal protection effect of the PermaNet^®^ Dual found in this study are the desired outcomes of any vector control tool. The high mortality effects and the additive blood-feeding inhibition impacts induced by PermaNet^®^ Dual in this hut trial are expected to substantially diminish the malaria vector density and biting rates in the field, and hence the transmission of malaria in the areas where vectors are resistant to insecticides [40–47]. Additionally, PermaNet^®^ Dual performed better than the reference PermaNet^®^ 3.0 and PermaNet^®^ 2.0 nets, when washed 20 times, thus meeting the WHO hut trial criteria [23].

However, these results need to be further validated in a large-scale field trial to assess the durability and acceptability of this new tool for malaria vector control [48–50]. A community trial would be the best way to evaluate the community level effect of this promising candidate net (i.e. PermaNet^®^ Dual) against malaria transmission, by monitoring entomological infection rate, *Plasmodium* prevalence and disease incidence [48–50]. Such a community trial could be a randomized controlled trial as with dual-active-ingredient LLINs (e.g. Interceptor G2) in Tanzania [49], soon in Benin [48, 50], and the pilot deployments that are part of the New Nets Project led by PATH [51]. Furthermore, PermaNet^®^ Dual should be tested against wild populations of *Anopheles funestus* [32], or other main or secondary vectors of malaria in Africa [52–56]. Ultimately, in the present study, the series of hut and laboratory tests demonstrated that the chlorfenapyr component of PermaNet^®^ Dual could make a major contribution to controlling the pyrethroid-resistant *An. gambiae* populations. Furthermore, there were no adverse effects reported among hut sleepers and mosquito collectors during the trial in the huts where PermaNet^®^ Dual were used, and this may possibly increase the rate of future user acceptability and improve the usage of this net for malaria vector control.

## Conclusion

The present small-scale GLP experimental hut study demonstrated that the deltamethrin- chlorfenapyr PermaNet^®^ Dual net has high bio-efficacy, inducing significantly higher mortality and blood-feeding inhibition effects in the wild insecticide-resistant *An. gambiae s.l*. when compared with the deltamethrin-PBO PermaNet^®^ 3.0 and deltamethrin-only PermaNet^®^ 2.0. The inclusion of chlorfenapyr with pyrethroid in PermaNet^®^ Dual net has greatly improved protection and control of free-flying wild pyrethroid-resistant *An. gambiae* populations. The chlorfenapyr-deltamethrin-coated PermaNet^®^ Dual net has great potential to reduce malaria transmission, particularly in areas compromised by high level of pyrethroid resistance in *Anopheles* mosquitoes. Further validations in large-scale field trials are required to assess the effectiveness, the durability and the acceptability of this new tool for malaria vector control.

## Supplementary Information

**Additional file 1: Table S1.** Up to 72-h mortality rates in pyrethroid-resistant *Anopheles gambiae s.l.* in experimental hut trials with long-lasting insecticidal nets in Tiassalé, southern Côte d'Ivoire.

**Additional file 2: Table S2.** Mean knock-down and mortality rates in multi-resistant *Anopheles gambiae s.s.* (Kisumu strain) exposed to long-lasting insecticidal nets using cone bioassays before and after experimental hut trial in Tiassalé, Côte d’Ivoire.

**Additional file 3: Table S3.** Mean knock-down and mortality rates in multi-resistant *Anopheles gambiae s.l.* (Tiassalé strain) exposed to long-lasting insecticidal nets using cone bioassays before and after experimental hut trial in Tiassalé, Côte d’Ivoire.

**Additional file 4: Table S4.** Up to 72-h mortality rates in susceptible populations of *Anopheles gambiae s.s.* (Kisumu strain) exposed to long-lasting insecticidal nets using tunnel tests before and after the experimental hut trial.

**Additional file 5: Table S5.** Up to 72-h mortality in multiple-resistant populations of *Anopheles gambiae s.l.* (Tiassalé strain) exposed to long-lasting insecticidal nets using tunnel tests before and after the experimental hut trial.

## Data Availability

All relevant data are within the manuscript.

## Abbreviations

WHO: World Health Organization
LLIN: Long-lasting insecticidal net
GPIRM: Global Plan for Insecticide Resistance Management
GTS: Global technical strategy
s.l.: Sensu lato
s.s.: Sensu stricto
MFO: Mixed function oxidase
GST: Glutathione S-transferase
CDC: Centers for Disease Control and Prevention
PBO: Piperonyl butoxide
GLP: Good laboratory practice
PQT/VCP: Prequalification Unit, Vector Control Product Assessment Team
CIPAC: Collaborative International Pesticides Analytical Council
GLMM: Generalized linear mixed model
SEM: Standard error of the mean
