# Supplementary material for: Small-scale field evaluation of PermaNet® Dual (a long-lasting net coated with a mixture of chlorfenapyr and deltamethrin) against pyrethroid-resistant Anopheles gambiae mosquitoes from Tiassalé, Côte d’Ivoire
Source: Malar J. 2023 Feb 1;22:36. doi: 10.1186/s12936-023-04455-z (PMC9893697; doi:10.1186/s12936-023-04455-z)
Supplement: Supplementary file 1 — Additional file 1: Table S1. Up to 72-h mortality rates in pyrethroid-resistant Anopheles gambiae s.l. in experimental hut trials with long-lasting insecticidal nets in Tiassalé, southern Côte d'Ivoire. [file 12936_2023_4455_MOESM1_ESM.docx]

| **Additional file 1: Table S1.** Up to 72-hour mortality rates in pyrethroid-resistant *Anopheles gambiae* s.l. in experimental hut trials with long-lasting insecticidal nets in Tiassalé, southern Côte d'Ivoire | | | | | | | | | |
| --- | --- | --- | --- | --- | --- | --- | --- | --- | --- |
| **Parameter** | **Summary data** | **Untreated net (control)** | **PermaNet^®^ Dual (A) unwashed** | **PermaNet^®^ Dual (B) unwashed** | **PermaNet^®^ Dual (B) washed** | **PermaNet^®^ 3.0 unwashed** | **PermaNet^®^ 3.0 washed** | **PermaNet^®^ 2.0 unwashed** | **PermaNet^®^ 2.0 washed** |
| Total number | Total number of females caught | 519 | 421 | 381 | 612 | 326 | 558 | 381 | 669 |
| Immediate mortality | Number of dead females morning (immediate mortality) | 1 | 372 | 338 | 509 | 153 | 157 | 56 | 133 |
|  | Immediate mortality (%): mean ± SEM | 0.0 ± 0.4 | 77.1 ± 1.3 | 72.4 ± 1.4 | 82.8 ± 0.4 | 31.9 ± 3.5 | 10.6 ± 4.3 | 5.0 ± 4.7 | 7.4 ± 3.8 |
|  | Immediate mortality corrected for control (%): mean ± SEM | 0 | 77.1 ± 1.3 | 72.4 ± 1.4 | 82.8 ± 0.4 | 31.8 ± 3.5 | 10.4 ± 4.3 | 5.0 ± 4.7 | 7.4 ± 3.8 |
| 24-hour mortality | Number of dead females after 24 h (delayed mortality) | 1 | 14 | 11 | 33 | 10 | 22 | 13 | 24 |
|  | Total number of dead females after 24 hours | 2 | 386 | 349 | 542 | 163 | 179 | 69 | 157 |
|  | 24-hour mortality (%): mean ± SEM | 0.6 ± 0.3 | 92.5 ± 0.2 | 82.9 ± 0.9 | 88.2 ± 0.2 | 37.4 ± 2.9 | 14.2 ± 3.9 | 7.2 ± 5.0 | 10.3 ± 3.6 |
|  | 24-hour mortality corrected for control (%): mean ± SEM | 0.0 | 92.5 ± 0.2 | 82.8 ± 0.9 | 88.2 ± 0.2 | 37.4 ± 2.9 | 13.8 ± 3.9 | 7.2 ± 5.0 | 9.9 ± 3.7 |
| 48-hour mortality | Number of dead females after 48 hours (delayed mortality) | 2 | 17 | 13 | 34 | 10 | 27 | 14 | 30 |
|  | Total number of dead females after 48 hours | 3 | 389 | 351 | 543 | 163 | 184 | 70 | 163 |
|  | 48-hour mortality (%): mean ± SEM | 0.1 ± 0.7 | 93.4 ± 0.2 | 83.3 ± 0.9 | 88.5 ± 0.2 | 37.4 ± 2.9 | 14.5 ± 3.9 | 7.5 ± 5.0 | 10.7 ± 3.7 |
|  | 48-hour mortality corrected for control (%): mean ± SEM | 0.0 | 93.4 ± 0.2 | 83.2 ± 0.9 | 88.4 ± 0.2 | 37.3 ± 2.9 | 14.0 ± 3.9 | 7.5 ± 5.0 | 10.5 ± 3.6 |
| 72-hour mortality | Number of dead females after 72 hours (delayed mortality) | 2 | 20 | 13 | 35 | 11 | 32 | 16 | 34 |
|  | Total number of dead females after 72 hours | 3 | 392 | 351 | 544 | 164 | 189 | 72 | 167 |
|  | 72-hour mortality (%): mean ± SEM | 0.1 ± 0.7 | 93.6 ± 0.2 | 83.3 ± 0.9 | 88.7 ± 0.2 | 37.5 ± 2.9 | 14.8 ± 3.9 | 7.4 ± 5.1 | 11.9 ± 3.4 |
|  | 72-hour mortality corrected for control (%): mean ± SEM | 0.0 | 93.6 ± 0.2 | 83.2 ± 0.9 | 88.7 ± 0.2 | 37.5 ± 2.9 | 14.4 ± 3.9 | 7.4 ± 5.1 | 11.7 ± 3.4 |
| %: percentage, h: hour, SEM: standard error of the mean. Each washed net sample was washed 20 times. | | | | | | | | | |
