# Supplementary material for: Small-scale field evaluation of PermaNet® Dual (a long-lasting net coated with a mixture of chlorfenapyr and deltamethrin) against pyrethroid-resistant Anopheles gambiae mosquitoes from Tiassalé, Côte d’Ivoire
Source: Malar J. 2023 Feb 1;22:36. doi: 10.1186/s12936-023-04455-z (PMC9893697; doi:10.1186/s12936-023-04455-z)
Supplement: Supplementary file 2 — Additional file 2: Table S2. Mean knock-down and mortality rates in multi-resistant Anopheles gambiae s.s. (Kisumu strain) exposed to long-lasting insecticidal nets using cone bioassays before and after experimental hut trial in Tiassalé, Côte d’Ivoire. [file 12936_2023_4455_MOESM2_ESM.docx]

| **Additional file 2: Table S2** Mean knock-down and mortality rates in multi-resistant *Anopheles gambiae* s.s. (Kisumu strain) exposed to long-lasting insecticidal nets using cone bioassays before and after experimental hut trial in Tiassalé, Côte d’Ivoire | | | | | | | | | |
| --- | --- | --- | --- | --- | --- | --- | --- | --- | --- |
| **Parameter** | **Summary data** | **Untreated net (control)** | **PermaNet^®^ Dual (A) unwashed** | **PermaNet^®^ Dual (B) unwashed** | **PermaNet^®^ Dual (B) washed** | **PermaNet^®^ 3.0 unwashed** | **PermaNet^®^ 3.0 washed** | **PermaNet^®^ 2.0 unwashed** | **PermaNet^®^ 2.0 washed** |
| **Before hut trial** |  |  |  |  |  |  |  |  |  |
| 24-hour mortality | Number dead females after 24 h | 1 | 35 | 36 | 50 | 50 | 47 | 48 | 48 |
|  | Number alive females after 24 h | 49 | 15 | 14 | 0 | 0 | 3 | 2 | 2 |
|  | 24-h mortality rate: mean ± SEM (%) | 2.0 ± 0.0 | 70.0 ± 7.1 | 72.0 ± 6.6 | 100.0 ± 0.0 | 100.0 ± 0.0 | 94.0 ± 2.4 | 96.0 ± 4.0 | 96.0 ± 4.0 |
|  | 24-h mortality corrected for control: mean ± SEM (%) |  | 70.0 ± 7.1 | 72.0 ± 6.6 | 100.0 ± 0.0 | 100.0 ± 0.0 | 94.0 ± 2.4 | 96.0 ± 4.0 | 96.0 ± 4.0 |
| 48-hour mortality | Number dead females after 48 h | 0 | 35 | 37 | 50 | 50 | 49 | 49 | 50 |
|  | Number alive females after 48 h | 50 | 15 | 13 | 0 | 0 | 1 | 1 | 0 |
|  | 48-h mortality rate: mean ± SEM (%) | 0.0 ± 0.0 | 70.0 ± 7.1 | 74.0 ± 6.8 | 100.0 ± 0.0 | 100.0 ± 0.0 | 98.0 ± 2.0 | 98.0 ± 2.0 | 100.0 ± 0.0 |
|  | 48-h mortality corrected for control: mean ± SEM (%) | 0 | 70.0 ± 7.1 | 76.0 ± 8.1 | 100.0 ± 0.0 | 100.0 ± 0.0 | 98.0 ± 2.0 | 98.0 ± 2.0 | 100.0 ± 0.0 |
| 72-hour mortality | Number dead females after 72 h | 1 | 35 | 38 | 50 | 50 | 50 | 50 | 50 |
|  | Number alive females after 72 h | 49 | 15 | 12 | 0 | 0 | 0 | 0 | 0 |
|  | 72-h mortality rate: mean ± SEM (%) | 2.0 ± 0.0 | 70.0 ± 7.1 | 76.0 ± 8.1 | 100.0 ± 0.0 | 100.0 ± 0.0 | 100.0 ± 0.0 | 100.0 ± 0.0 | 100.0 ± 0.0 |
|  | 72-h mortality corrected for control: mean ± SEM (%) |  | 70.0 ± 7.1 | 76.0 ± 8.1 | 100.0 ± 0.0 | 100.0 ± 0.0 | 100.0 ± 0.0 | 100.0 ± 0.0 | 100.0 ± 0.0 |
| **After hut trial** |  |  |  |  |  |  |  |  |  |
| 24-hour mortality | Number dead females after 24 h | 0 | 17 | 35 | 44 | 47 | 41 | 42 | 24 |
|  | Number alive females after 24 h | 50 | 33 | 15 | 6 | 3 | 9 | 8 | 26 |
|  | 24-h mortality rate: mean ± SEM (%) | 0.0 ± 0.0 | 34.0 ± 7.5 | 70.0 ± 8.4 | 88.0 ± 5.8 | 94.0 ± 8.9 | 82.0 ± 12.0 | 84.0 ± 5.1 | 48.0 ± 9.7 |
|  | 24-h mortality corrected for control: mean ± SEM (%) | 0 | 34.0 ± 7.5 | 70.0 ± 8.4 | 88.0 ± 5.8 | 94.0 ± 8.9 | 82.0 ± 12.0 | 84.0 ± 5.1 | 48.0 ± 9.7 |
| 48-hour mortality | Number dead females after 48 h | 0 | 17 | 43 | 44 | 47 | 42 | 44 | 25 |
|  | Number alive females after 48 h | 50 | 33 | 7 | 6 | 3 | 8 | 6 | 25 |
|  | 48-h mortality rate: mean ± SEM (%) | 0.0 ± 0.0 | 34.0 ± 7.5 | 86.0 ± 5.1 | 88.0 ± 5.8 | 94 ± 4.0 | 84.0 ± 11.7 | 88.0 ± 3.7 | 50.0 ± 10.5 |
|  | 48-h mortality corrected for control: mean ± SEM (%) | 0 | 34.0 ± 7.5 | 86.0 ± 5.1 | 88.0 ± 5.8 | 94 ± 4.0 | 84.0 ± 11.7 | 88.0 ± 3.7 | 50.0 ± 10.5 |
| 72-hour mortality | Number dead females after 72 h | 0 | 17 | 43 | 47 | 50 | 44 | 50 | 30 |
|  | Number alive females after 72 h | 50 | 33 | 7 | 3 | 0 | 6 | 0 | 20 |
|  | 72-h mortality rate: mean ± SEM (%) | 0.0 ± 0.0 | 34.0 ± 7.5 | 86.0 ± 5.1 | 94.0 ± 6.0 | 100.0 ± 0.0 | 88.0 ± 12.0 | 100.0 ± 0.0 | 60.0 ± 16.4 |
|  | 72-h mortality corrected for control: mean ± SEM (%) | 0 | 34.0 ± 7.5 | 86.0 ± 5.1 | 94.0 ± 6.0 | 100.0 ± 0.0 | 88.0 ± 12.0 | 100.0 ± 0.0 | 60.0 ± 16.4 |
| %: percentage, min: minute, KD60: knock-down, KD: knock-down after 60 min, h: hour, SEM: standard error of the mean. Each washed net sample was washed 20 times. A total number of 50 females of *An. gambiae* s.s. Kisumu strain were tested per net sample. | | | | | | | | | |
