# Supplementary material for: Small-scale field evaluation of PermaNet® Dual (a long-lasting net coated with a mixture of chlorfenapyr and deltamethrin) against pyrethroid-resistant Anopheles gambiae mosquitoes from Tiassalé, Côte d’Ivoire
Source: Malar J. 2023 Feb 1;22:36. doi: 10.1186/s12936-023-04455-z (PMC9893697; doi:10.1186/s12936-023-04455-z)
Supplement: Supplementary file 3 — Additional file 3: Table S3. Mean knock-down and mortality rates in multi-resistant Anopheles gambiae s.l. (Tiassalé strain) exposed to long-lasting insecticidal nets using cone bioassays before and after experimental hut trial in Tiassalé, Côte d’Ivoire. [file 12936_2023_4455_MOESM3_ESM.docx]

| **Additional file 3: Table S3.** Mean knock-down and mortality rates in multi-resistant *Anopheles gambiae* s.l. (Tiassalé strain) exposed to long-lasting insecticidal nets using cone bioassays before and after experimental hut trial in Tiassalé, Côte d’Ivoire | | | | | | | | | |
| --- | --- | --- | --- | --- | --- | --- | --- | --- | --- |
| **Parameter** | **Summary data** | **Untreated net (control)** | **PermaNet^®^ Dual (A) unwashed** | **PermaNet^®^ Dual (B) unwashed** | **PermaNet^®^ Dual (B) washed** | **PermaNet^®^ 3.0 unwashed** | **PermaNet^®^ 3.0 washed** | **PermaNet^®^ 2.0 unwashed** | **PermaNet^®^ 2.0 washed** |
| **Before hut trial** |  |  |  |  |  |  |  |  |  |
| 24-hour mortality | Number dead females after 24 h | 0 | 7 | 9 | 15 | 23 | 8 | 18 | 7 |
|  | Number alive females after 24 h | 50 | 43 | 41 | 35 | 27 | 42 | 32 | 43 |
|  | 24-h mortality rate: mean ± SEM (%) | 0.0 ± 0.0 | 14.0 ± 6.8 | 18.0 ± 8.0 | 30.0 ± 3.7 | 46.0 ± 16.9 | 16.0 ± 2.4 | 36.0 ± 9.3 | 14.0 ± 4.0 |
|  | 24-h mortality corrected for control: mean ± SEM (%) | 0 | 14.0 ± 6.8 | 18.0 ± 8.0 | 30.0 ± 3.7 | 46.0 ± 16.9 | 16.0 ± 2.4 | 36.0 ± 9.3 | 14.0 ± 4.0 |
| 48-hour mortality | Number dead females after 48 h | 0 | 7 | 12 | 18 | 23 | 8 | 20 | 8 |
|  | Number alive females after 48 h | 50 | 43 | 38 | 32 | 27 | 42 | 30 | 42 |
|  | 48-h mortality rate: mean ± SEM (%) | 0.0 ± 0.0 | 14.0 ± 6.8 | 24.0 ± 11.2 | 36.0 ± 6.8 | 46.0 ± 16.9 | 16.0 ± 2.4 | 40.0 ± 13.0 | 16.0 ± 5.1 |
|  | 48-h mortality corrected for control: mean ± SEM (%) | 0 | 14.0 ± 6.8 | 24.0 ± 11.2 | 36.0 ± 6.8 | 46.0 ± 16.9 | 16.0 ± 2.4 | 40.0 ± 13.0 | 16.0 ± 5.1 |
| 72-hour mortality | Number dead females after 72 h | 0 | 7 | 12 | 19 | 24 | 9 | 21 | 8 |
|  | Number alive females after 72 h | 50 | 43 | 38 | 31 | 26 | 41 | 29 | 42 |
|  | 72-h mortality rate: mean ± SEM (%) | 0.0 ± 0.0 | 14.0 ± 6.8 | 24.0 ± 11.2 | 38.0 ± 7.0 | 48.0 ± 16.6 | 18.0 ± 3.7 | 42.0 ± 13.2 | 16.0 ± 5.1 |
|  | 72-h mortality corrected for control: mean ± SEM (%) | 0 | 14.0 ± 6.8 | 24.0 ± 11.2 | 38.0 ± 7.0 | 48.0 ± 16.6 | 18.0 ± 3.7 | 42.0 ± 13.2 | 16.0 ± 5.1 |
| **After hut trial** |  |  |  |  |  |  |  |  |  |
| 24-hour mortality | Number dead females after 24 h | 0 | 6 | 8 | 4 | 11 | 6 | 12 | 9 |
|  | Number alive females after 24 h | 50 | 44 | 42 | 46 | 39 | 44 | 38 | 41 |
|  | 24-h mortality rate: mean ± SEM (%) | 0.0 ± 0.0 | 12.0 ± 4.9 | 16.0 ± 6.0 | 8.0 ± 4.5 | 22.0 ± 3.7 | 12.0 ± 2.0 | 24.0 ± 7.5 | 18.0 ± 5.8 |
|  | 24-h mortality corrected for control: mean ± SEM (%) | 0 | 12.0 ± 4.9 | 16.0 ± 6.0 | 8.0 ± 4.5 | 22.0 ± 3.7 | 12.0 ± 2.0 | 24.0 ± 7.5 | 18.0 ± 5.8 |
| 48-hour mortality | Number dead females after 48 h | 0 | 6 | 8 | 5 | 11 | 9 | 14 | 12 |
|  | Number alive females after 48 h | 50 | 44 | 42 | 45 | 39 | 41 | 36 | 38 |
|  | 48-h mortality rate: mean ± SEM (%) | 0.0 ± 0.0 | 12.0 ± 4.9 | 16.0 ± 6.0 | 10.0 ± 3.2 | 22.0 ± 3.7 | 18.0 ± 3.7 | 28 ± 8.6 | 24.0 ± 7.5 |
|  | 48-h mortality corrected for control: mean ± SEM (%) | 0 | 12.0 ± 4.9 | 16.0 ± 6.0 | 10.0 ± 3.2 | 22.0 ± 3.7 | 18.0 ± 3.7 | 28 ± 8.6 | 24.0 ± 7.5 |
| 72-hour mortality | Number dead females after 72 h | 0 | 7 | 12 | 5 | 11 | 9 | 14 | 12 |
|  | Number alive females after 72 h | 50 | 43 | 38 | 45 | 39 | 41 | 36 | 38 |
|  | 72-h mortality rate: mean ± SEM (%) | 0.0 ± 0.0 | 14.0 ± 6.0 | 24.0 ± 10.3 | 10.0 ± 3.2 | 22.0 ± 3.7 | 18.0 ± 3.7 | 28.0 ± 8.6 | 24.0 ± 7.4 |
|  | 72-h mortality corrected for control: mean ± SEM (%) | 0 | 14.0 ± 6.0 | 24.0 ± 10.3 | 10.0 ± 3.2 | 22.0 ± 3.7 | 18.0 ± 3.7 | 28.0 ± 8.6 | 24.0 ± 7.4 |
| %: percentage, min: minute, KD60: knock-down, KD: knock-down after 60 min, h: hour, SEM: standard error of the mean. Each washed net sample was washed 20 times. A total number of 50 females of *An. gambiae* s.l. Tiassalé strain were tested per net sample. | | | | | | | | | |
