# Supplementary material for: Small-scale field evaluation of PermaNet® Dual (a long-lasting net coated with a mixture of chlorfenapyr and deltamethrin) against pyrethroid-resistant Anopheles gambiae mosquitoes from Tiassalé, Côte d’Ivoire
Source: Malar J. 2023 Feb 1;22:36. doi: 10.1186/s12936-023-04455-z (PMC9893697; doi:10.1186/s12936-023-04455-z)
Supplement: Supplementary file 4 — Additional file 4: Table S4. Up to 72-h mortality rates in susceptible populations of Anopheles gambiae s.s. (Kisumu strain) exposed to long-lasting insecticidal nets using tunnel tests before and after the experimental hut trial. [file 12936_2023_4455_MOESM4_ESM.docx]

| **Additional file 4: Table S4.** Up to 72-hour mortality rates in susceptible populations of *Anopheles gambiae* s.s. (Kisumu strain) exposed to long-lasting insecticidal nets using tunnel tests before and after the experimental hut trial | | | | | | | | | |
| --- | --- | --- | --- | --- | --- | --- | --- | --- | --- |
| **Parameter** | **Summary data** | **Untreated net (control)** | **PermaNet^®^ Dual (A) unwashed** | **PermaNet^®^ Dual (B) unwashed** | **PermaNet^®^ Dual (B) washed** | **PermaNet^®^ 3.0 unwashed** | **PermaNet^®^ 3.0 washed** | **PermaNet^®^ 2.0 unwashed** | **PermaNet^®^ 2.0 washed** |
| **After hut trial** |  |  |  |  |  |  |  |  |  |
| Immediate (15-hour) mortality | Number of dead females after 15 h | 26 | 500 | 500 | 500 | 500 | 500 | 500 | 500 |
|  | Number of alive females after 15 h | 674 | 0 | 0 | 0 | 0 | 0 | 0 | 0 |
|  | 15-h mortality rate: mean ± SEM (%) | 3.7 ± 0.2 | 100.0 ± 0.0 | 100.0 ± 0.0 | 100.0 ± 0.0 | 100.0 ± 0.0 | 100.0 ± 0.0 | 100.0 ± 0.0 | 100.0 ± 0.0 |
|  | 15-h mortality corrected for control: mean ± SEM (%) | 0 | 100.0 ± 0.0 | 100.0 ± 0.0 | 100.0 ± 0.0 | 100.0 ± 0.0 | 100.0 ± 0.0 | 100.0 ± 0.0 | 100.0 ± 0.0 |
| 24-hour mortality | Number of dead females after 24 h | 26 | 500 | 500 | 500 | 500 | 500 | 500 | 500 |
|  | Number of alive females after 24 h | 674 | 0 | 0 | 0 | 0 | 0 | 0 | 0 |
|  | 24-h mortality rate: mean ± SEM (%) | 3.7 ± 0.2 | 100.0 ± 0.0 | 100.0 ± 0.0 | 100.0 ± 0.0 | 100.0 ± 0.0 | 100.0 ± 0.0 | 100.0 ± 0.0 | 100.0 ± 0.0 |
|  | 24-h mortality corrected for control: mean ± SEM (%) | 0 | 100.0 ± 0.0 | 100.0 ± 0.0 | 100.0 ± 0.0 | 100.0 ± 0.0 | 100.0 ± 0.0 | 100.0 ± 0.0 | 100.0 ± 0.0 |
| 48-hour mortality | Number of dead females after 48 h | 26 | 500 | 500 | 500 | 500 | 500 | 500 | 500 |
|  | Number of alive females after 48 h | 674 | 0 | 0 | 0 | 0 | 0 | 0 | 0 |
|  | 48-h mortality rate: mean ± SEM (%) | 3.7 ± 0.2 | 100.0 ± 0.0 | 100.0 ± 0.0 | 100.0 ± 0.0 | 100.0 ± 0.0 | 100.0 ± 0.0 | 100.0 ± 0.0 | 100.0 ± 0.0 |
|  | 48-h mortality corrected for control: mean ± SEM (%) | 0 | 100.0 | 100.0 | 100.0 | 100.0 | 100.0 | 100.0 | 100.0 |
| 72-hour mortality | Number of dead females after 72 h | 26 | 500 | 500 | 500 | 500 | 500 | 500 | 500 |
|  | Number of alive females after 72 h | 674 | 0 | 0 | 0 | 0 | 0 | 0 | 0 |
|  | 72-h mortality rate: mean ± SEM (%) | 3.7 ± 0.2 | 100.0 ± 0.0 | 100.0 ± 0.0 | 100.0 ± 0.0 | 100.0 ± 0.0 | 100.0 ± 0.0 | 100.0 ± 0.0 | 100.0 ± 0.0 |
|  | 72-h mortality corrected for control: mean ± SEM (%) | 0 | 100.0 ± 0.0 | 100.0 ± 0.0 | 100.0 ± 0.0 | 100.0 ± 0.0 | 100.0 ± 0.0 | 100.0 ± 0.0 | 100.0 ± 0.0 |
| **After hut trial** |  |  |  |  |  |  |  |  |  |
| Immediate (15-hour) mortality | Number of dead females after 15 h | 8 | 500 | 500 | 500 | 500 | 500 | 500 | 500 |
|  | Number of alive females after 15 h | 692 | 0 | 0 | 0 | 0 | 0 | 0 | 0 |
|  | 15-h mortality rate: mean ± SEM (%) | 1.1 ± 0.3 | 100.0 ± 0.0 | 100.0 ± 0.0 | 100.0 ± 0.0 | 100.0 ± 0.0 | 100.0 ± 0.0 | 100.0 ± 0.0 | 100.0 ± 0.0 |
|  | 15-h mortality corrected for control: mean ± SEM (%) | 0 | 100.0 ± 0.0 | 100.0 ± 0.0 | 100.0 ± 0.0 | 100.0 ± 0.0 | 100.0 ± 0.0 | 100.0 ± 0.0 | 100.0 ± 0.0 |
| 24-hour mortality | Number of dead females after 24 h | 8 | 500 | 500 | 500 | 500 | 500 | 500 | 500 |
|  | Number of alive females after 24 h | 692 | 0 | 0 | 0 | 0 | 0 | 0 | 0 |
|  | 24-h mortality rate: mean ± SEM (%) | 1.1 ± 0.3 | 100.0 ± 0.0 | 100.0 ± 0.0 | 100.0 ± 0.0 | 100.0 ± 0.0 | 100.0 ± 0.0 | 100.0 ± 0.0 | 100.0 ± 0.0 |
|  | 24-h mortality corrected for control: mean ± SEM (%) | 0 | 100.0 ± 0.0 | 100.0 ± 0.0 | 100.0 ± 0.0 | 100.0 ± 0.0 | 100.0 ± 0.0 | 100.0 ± 0.0 | 100.0 ± 0.0 |
| 48-hour mortality | Number of dead females after 48 h | 8 | 500 | 500 | 500 | 500 | 500 | 500 | 500 |
|  | Number of alive females after 48 h | 692 | 0 | 0 | 0 | 0 | 0 | 0 | 0 |
|  | 48-h mortality rate: mean ± SEM (%) | 1.1 ± 0.3 | 100.0 ± 0.0 | 100.0 ± 0.0 | 100.0 ± 0.0 | 100.0 ± 0.0 | 100.0 ± 0.0 | 100.0 ± 0.0 | 100.0 ± 0.0 |
|  | 48-h mortality corrected for control: mean ± SEM (%) | 0 | 100.0 ± 0.0 | 100.0 ± 0.0 | 100.0 ± 0.0 | 100.0 ± 0.0 | 100.0 ± 0.0 | 100.0 ± 0.0 | 100.0 ± 0.0 |
| 72-hour mortality | Number of dead females after 72 h | 8 | 500 | 500 | 500 | 500 | 500 | 500 | 500 |
|  | Number of alive females after 72 h | 692 | 0 | 0 | 0 | 0 | 0 | 0 | 0 |
|  | 72-h mortality rate: mean ± SEM (%) | 1.1 ± 0.3 | 100.0 ± 0.0 | 100.0 ± 0.0 | 100.0 ± 0.0 | 100.0 ± 0.0 | 100.0 ± 0.0 | 100.0 ± 0.0 | 100.0 ± 0.0 |
|  | 72-h mortality corrected for control: mean ± SEM (%) | 0 | 100.0 ± 0.0 | 100.0 ± 0.0 | 100.0 ± 0.0 | 100.0 ± 0.0 | 100.0 ± 0.0 | 100.0 ± 0.0 | 100.0 ± 0.0 |
| %: percentage, h: hour, SEM: standard error of the mean. Each washed net sample was washed 20 times. A total number of 500 females of *An. gambiae* s.s. Kisumu strain were tested per net sample. | | | | | | | | | |
