# Supplementary material for: Small-scale field evaluation of PermaNet® Dual (a long-lasting net coated with a mixture of chlorfenapyr and deltamethrin) against pyrethroid-resistant Anopheles gambiae mosquitoes from Tiassalé, Côte d’Ivoire
Source: Malar J. 2023 Feb 1;22:36. doi: 10.1186/s12936-023-04455-z (PMC9893697; doi:10.1186/s12936-023-04455-z)
Supplement: Supplementary file 5 — Additional file 5: Table S5. Up to 72-h mortality in multiple-resistant populations of Anopheles gambiae s.l. (Tiassalé strain) exposed to long-lasting insecticidal nets using tunnel tests before and after the experimental hut trial. [file 12936_2023_4455_MOESM5_ESM.docx]

| **Additional file 5: Table S5.** Up to 72-hour mortality in multiple-resistant populations of *Anopheles gambiae* s.l. (Tiassalé strain) exposed to long-lasting insecticidal nets using tunnel tests before and after the experimental hut trial | | | | | | | | | |
| --- | --- | --- | --- | --- | --- | --- | --- | --- | --- |
| **Post-exposure time (h)** | **Summary data** | **Untreated net (control)** | **PermaNet^®^ Dual (A) unwashed** | **PermaNet^®^ Dual (B) unwashed** | **PermaNet^®^ Dual (B) washed** | **PermaNet^®^ 3.0 unwashed** | **PermaNet^®^ 3.0 washed** | **PermaNet^®^ 2.0 unwashed** | **PermaNet^®^ 2.0 washed** |
| **After hut trial** |  |  |  |  |  |  |  |  |  |
| Immediate (15-hour) mortality | Number of dead females after 15 h | 25 | 376 | 263 | 313 | 372 | 354 | 193 | 219 |
|  | Number of alive females after 15 h | 675 | 124 | 237 | 187 | 128 | 146 | 307 | 281 |
|  | 15-h mortality rate: mean ± SEM (%) | 3.6 ± 0.4 | 75.2 ± 4.0 | 52.6 ± 3.1 | 62.6 ± 4.2 | 74.4 ± 6.5 | 70.8 ± 7.2 | 38.6 ± 5.4 | 43.8 ± 2.5 |
|  | 15-h mortality corrected for control: mean ± SEM (%) |  | 74.4 ± 4.1 | 50.1 ± 3.3 | 60.6 ± 4.4 | 73.6 ± 6.7 | 69.9 ± 7.4 | 36.7 ± 5.6 | 42.1 ± 2.6 |
| 24-hour mortality | Number of dead females after 24 h | 25 | 464 | 412 | 422 | 416 | 395 | 245 | 280 |
|  | Number of alive females after 24 h | 675 | 36 | 88 | 78 | 84 | 105 | 255 | 220 |
|  | 24-h mortality rate: mean ± SEM (%) | 3.6 ± 0.4 | 92.8 ± 2.1 | 82.4 ± 3.9 | 84.4 ± 4.5 | 83.2 ± 5.1 | 79.0 ± 5.6 | 49.0 ± 3.6 | 56 .0 ± 1.8 |
|  | 24-h mortality corrected for control: mean ± SEM (%) | 0 | 92.6 ± 2.2 | 81.5 ± 4.1 | 83.6 ± 4.7 | 82.7 ± 5.3 | 78.4 ± 5.7 | 47.4 ± 3.7 | 54.6 ± 1.9 |
| 48-hour mortality | Number of dead females after 48 h | 25 | 467 | 427 | 427 | 419 | 397 | 245 | 280 |
|  | Number of alive females after 48 h | 675 | 33 | 73 | 73 | 81 | 103 | 255 | 220 |
|  | 48-h mortality rate: mean ± SEM (%) | 3.6 ± 0.4 | 93.4 ± 1.8 | 85.4 ± 3.9 | 85.4 ± 4.5 | 83.8 ± 5.1 | 79.4 ± 5.6 | 49.0 ± 3.6 | 56.0 ± 1.8 |
|  | 48-h mortality corrected for control: mean ± SEM (%) | 0 | 93.2 ± 1.9 | 84.6 ± 4.1 | 84.6 ± 4.7 | 83.3 ± 5.3 | 78.8 ± 5.8 | 47.4 ± 3.7 | 54.6 ± 1.9 |
| 72-hour mortality | Number of dead females after 72 h | 25 | 475 | 430 | 431 | 421 | 397 | 249 | 287 |
|  | Number of alive females after 72 h | 675 | 25 | 70 | 69 | 79 | 103 | 251 | 213 |
|  | 72-h mortality rate: mean ± SEM (%) | 3.6 ± 0.4 | 95.0 ± 1.3 | 86.0 ± 4.1 | 86.2 ± 4.7 | 84.2 ± 5.1 | 79.4 ± 5.6 | 49.8 ± 3.8 | 57.4 ± 1.8 |
|  | 72-h mortality corrected for control: mean ± SEM (%) | 0 | 94.9 ± 1.3 | 85.2 ± 4.4 | 85.5 ± 5.0 | 83.7 ± 5.2 | 78.8 ± 5.8 | 48.3 ± 3.9 | 56.1 ± 1.9 |
| **After hut trial** |  |  |  |  |  |  |  |  |  |
| Immediate (15-hour) mortality | Number of dead females after 15 h | 15 | 282 | 308 | 366 | 295 | 272 | 184 | 250 |
|  | Number of alive females after 15 h | 685 | 218 | 192 | 134 | 205 | 228 | 316 | 250 |
|  | 15-h mortality rate: mean ± SEM (%) | 2.1 ± 0.6 | 56.4 ± 4.0 | 61.6 ± 4.3 | 73.2 ± 6.1 | 59.0 ± 8.7 | 54.4 ± 6.9 | 36.8 ± 3.9 | 50.0 ± 3.4 |
|  | 15-h mortality corrected for control: mean ± SEM (%) | 0.0 | 55.1 ± 4.2 | 60.4 ± 4.5 | 72.1 ± 6.4 | 57.7 ±9.0 | 53.5 ± 7.1 | 36.8 ± 3.9 | 50.0 ± 3.4 |
| 24-hour mortality | Number of dead females after 24 h | 15 | 415 | 427 | 424 | 381 | 352 | 238 | 307 |
|  | Number of alive females after 24 h | 685 | 85 | 73 | 76 | 119 | 148 | 262 | 193 |
|  | 24-h mortality rate: mean ± SEM (%) | 2.1 ± 0.6 | 83.0 ± 5.0 | 85.4 ± 2.6 | 84.8 ± 2.5 | 76.2 ± 7.3 | 70.4 ± 5.7 | 47.6 ± 4.0 | 61.4 ± 4.7 |
|  | 24-h mortality corrected for control: mean ± SEM (%) | 0.0 | 82.5 ± 5.2 | 85.0 ± 2.7 | 84.2 ± 2.6 | 75.5 ± 7.5 | 69.8 ± 5.8 | 47.6 ± 4.0 | 61.4 ± 4.7 |
| 48-hour mortality | Number of dead females after 48 h | 16 | 424 | 434 | 437 | 383 | 352 | 239 | 309 |
|  | Number of alive females after 48 h | 685 | 76 | 66 | 63 | 117 | 148 | 261 | 191 |
|  | 48-h mortality rate: mean ± SEM (%) | 2.3 ± 0.6 | 84.8 ± 4.2 | 86.8 ± 2.6 | 87.4 ± 1.8 | 76.6 ± 7.3 | 70.4 ± 5.7 | 47.8 ± 3.9 | 61.8 ± 5.1 |
|  | 48-h mortality corrected for control: mean ± SEM (%) | 0.0 | 84.3 ± 4.3 | 86.3 ± 2.7 | 86.9 ± 1.9 | 75.9 ± 7.6 | 69.8 ± 5.8 | 47.8 ± 3.9 | 61.8 ± 5.1 |
| 72-hour mortality | Number of dead females after 72 h | 16 | 425 | 435 | 440 | 435 | 352 | 241 | 318 |
|  | Number of alive females after 72 h | 685 | 75 | 65 | 60 | 65 | 148 | 259 | 182 |
|  | 72-h mortality rate: mean ± SEM (%) | 2.3 ± 0.6 | 85.0 ± 4.2 | 87.0 ± 2.7 | 88.0 ± 1.5 | 79.6 ± 6.3 | 70.4 ± 5.7 | 48.2 ± 4.0 | 63.6 ± 4.7 |
|  | 72-h mortality corrected for control: mean ± SEM (%) | 0.0 | 84.5 ± 4.4 | 86.5 ± 2.8 | 87.5 ± 1.6 | 79.0 ± 6.4 | 69.8 ± 5.8 | 48.2 ± 4.0 | 63.6 ± 4.7 |
| %: percentage, h: hour, SEM: standard error of the mean. Each washed net sample was washed 20 times. A total number of 500 females of *An. gambiae* s.l. Tiassalé strain were tested per net sample. | | | | | | | | | |
